# Unveiling the Anti-Obesity Potential of Thunder God Vine: Network Pharmacology and Computational Insights into Celastrol-like Molecules

**DOI:** 10.3390/ijms252312501

**Published:** 2024-11-21

**Authors:** Siyun Zheng, Hengzheng Yang, Jingxian Zheng, Yidan Wang, Bo Jia, Wannan Li

**Affiliations:** 1 Key Laboratory for Molecular Enzymology and Engineering of Ministry of Education, School of Life Science, Jilin University, 2699 Qianjin Street, Changchun 130012, China; zhengsy1322@mails.jlu.edu.cn (S.Z.); yanghz24@mails.jlu.edu.cn (H.Y.); jxzheng24@mails.jlu.edu.cn (J.Z.); wangyd1322@mails.jlu.edu.cn (Y.W.); jiabo1322@mails.jlu.edu.cn (B.J.); 2 Edmond H. Fischer Signal Transduction Laboratory, School of Life Sciences, Jilin University, Changchun 130012, China

**Keywords:** network pharmacology, molecular docking, molecular dynamics simulations, celastrol-like molecules

## Abstract

Obesity, characterized by abnormal or excessive fat accumulation, has become a chronic degenerative health condition that poses significant threats to overall well-being. Pharmacological intervention stands at the forefront of strategies to combat this issue. Recent studies, notably by Umut Ozcan’s team, have uncovered the remarkable potential of Celastrol, a small-molecule compound derived from the traditional Chinese herb thunder god vine (Tripterygium wilfordii) as an anti-obesity agent. In this research, computational chemical analysis was employed, incorporating the “TriDimensional Hierarchical Fingerprint Clustering with Tanimoto Representative Selection (3DHFC-TRS)” algorithm to systematically explore 139 active small molecules from thunder god vine. These compounds were classified into six categories, with a particular focus on Category 1 molecules for their exceptional binding affinity to obesity-related targets, offering new avenues for therapeutic development. Using advanced molecular docking techniques and Cytoscape prediction models, six representative Celastrol-like molecules were identified, namely 3-Epikatonic Acid, Hederagenin, Triptonide, Triptotriterpenic Acid B, Triptotriterpenic Acid C, and Ursolic Acid. These compounds demonstrated superior binding affinity and specificity toward two key obesity targets, PPARG and PTGS2, suggesting their potential to regulate fat metabolism and mitigate inflammatory responses. To further substantiate these findings, molecular dynamics simulations and MM-PBSA free-energy calculations were applied to analyze the dynamic interactions between these small molecules and the enzymatic active sites of their targets. The results provide robust theoretical evidence that support the feasibility of these molecules as promising candidates for anti-obesity therapies. This study underscores the power of the 3DHFC-TRS algorithm in uncovering bioactive compounds from natural sources, such as thunder god vine, and highlights the therapeutic promise of PPARG and PTGS2 as novel obesity-related targets. Furthermore, it emphasizes the essential role of computational science in expediting drug discovery, paving the way for personalized and precision-based treatments for obesity and heralding a future of more effective healthcare solutions.

## 1. Introduction

In recent years, the global prevalence of obesity has surged significantly. The World Health Organization (WHO) has highlighted the gravity of this issue with alarming statistics. Since 1990, the global adult obesity rate has doubled, while adolescent obesity has more than doubled within just a few decades. This trend signals the likelihood of increasingly complex health challenges in the future [1]. Beyond its impact on physical appearance, obesity triggers a cascade of physiological responses, inducing chronic low-grade inflammation that contributes to the development of numerous chronic diseases [2].

The low-grade inflammation caused by obesity is closely associated with the development of type 2 diabetes [3,4]. During excessive adipocyte expansion, fat cells secrete various pro-inflammatory factors that impair normal insulin function, leading to dysregulated blood glucose control and, ultimately, the onset of diabetes. Moreover, obesity serves as an independent risk factor for hypertension [5] and hyperlipidemia [6], both of which contribute to cardiovascular diseases. Excessive body weight places additional strain on the heart [7], disrupts lipid metabolism, and promotes the development of atherosclerosis, posing serious threats to both health and life. Furthermore, it is estimated that 4% to 9% of cancer diagnoses are attributable to obesity, with strong correlations between obesity and poor outcomes in several malignant diseases [8,9].

Obesity is increasingly recognized as a chronic degenerative disease. Due to the limited effectiveness of lifestyle changes and behavioral interventions, pharmacological treatments or surgical procedures have become the most effective strategies for managing obesity. While bariatric surgery offers the most efficient approach, enabling rapid weight loss and reducing the incidence of comorbidities and mortality in obese individuals [10], it falls short of meeting global healthcare demands. As a result, pharmacological treatment has emerged as a preferred alternative in the fight against obesity [11]. The development of anti-obesity medications (AOMs) has been a complex journey. Early formulations had notable limitations, but recently approved drugs, such as orlistat [12], naltrexone/bupropion, liraglutide [13], and semaglutide [14,15], represent significant progress. However, these medications are not without drawbacks, as they have been linked to side effects such as hepatotoxicity, steatorrhea [16], abdominal discomfort, and even a heightened risk of pancreatic cancer [17]. Therefore, it is crucial to explore new drug compounds and therapeutic strategies to effectively manage obesity while minimizing its associated side effects.

Since its potential weight-loss properties were discovered in 2015, the thunder god vine from the Celastraceae family has garnered significant attention [18]. Thunder god vine contains a complex chemical profile, with over 160 identified compounds [19], primarily consisting of triterpenoids, sesquiterpenoids, and flavonoids. It exhibits diverse pharmacological activities, including anti-inflammatory, immunosuppressive, and anti-tumor effects, primarily through the modulation of inflammatory factors and signaling pathways [20]. Notably, it has shown exceptional efficacy in the treatment of lupus conditions [21] and diabetes [22]. Among its bioactive compounds, triterpenoids like Celastrol have shown promise in combating obesity by enhancing leptin sensitivity and leveraging anti-inflammatory properties [23,24]. However, the precise molecular targets of Celastrol in humans remain elusive, and its exact mechanism of action is not fully understood. The current findings are primarily based on animal studies, underscoring the need for further research to clarify how these compounds exert their weight-loss effects. As a natural plant with diverse chemical components and extensive pharmacological potential, the thunder god vine is poised to play a pivotal role in future drug development and clinical applications. A deeper exploration of its molecular mechanisms could unlock new therapeutic opportunities. The discovery of Celastrol, in particular, has opened new avenues for obesity treatment, highlighting the broader application potential of the thunder god vine to various medical fields.

In drug design, computational chemistry serves as an effective tool for identifying potential drug candidates. Various virtual screening techniques have been developed to accelerate drug discovery while reducing costs [25]. These techniques, such as molecular docking, pharmacophore modeling, and quantitative structure–activity relationship (QSAR) modeling, enable researchers to rapidly evaluate the bioactivity of numerous compounds through computer simulations [26]. These methods not only conserve laboratory resources but also significantly shorten the drug development process. By leveraging computational models, researchers can predict a compound’s activity in advance, allowing them to focus on the most promising candidates. However, despite the clear advantages of virtual screening, computational costs remain a major challenge, particularly for large-scale applications. Traditional methods often demand considerable resources and time, making high-throughput screening both difficult and costly. To overcome these limitations, the integration of multiple computational tools has become increasingly popular. Combining different computational approaches allows researchers to achieve high-throughput screening while maintaining accuracy. As a result, ongoing efforts are focused on developing more efficient computational tools to reduce costs and further enhance the drug discovery process.

**Figure 1:**
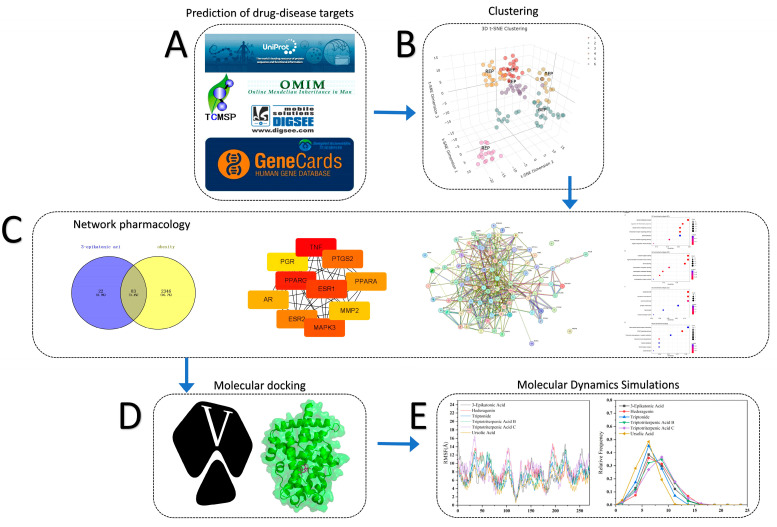
As illustrated in Figure 1, the network pharmacology workflow combines machine learning and quantitative computational methods to identify the key targets and related molecular mechanisms of the thunder god vine for the treatment of obesity. This process includes (**A**) Prediction of drug-disease targets, (**B**) Clustering, (**C**) Network pharmacology, (**D**) Molecular docking, (**E**) Molecular Dynamics Simulations.

In this study, the workflow is depicted in Figure 1. We retrieved 139 small molecules from the thunder god vine using the TCMSP and TCMID databases. These molecules underwent a clustering analysis using the TriDimensional Hierarchical Fingerprint Clustering with Tanimoto Representative Selection (3DHFC-TRS) algorithm. We then used UniProt to query the corresponding target protein gene names and conducted comparative analyses with obesity-related genes from OMIM, DigSee, and GeneCards. This approach ultimately led to the selection of six representative molecules, with a network pharmacology analysis confirming their association with obesity-related targets. A Venn diagram was used to identify the six small molecules with the most significant overlap between the target proteins and the obesity-related targets, which were then subjected to molecular dynamics (MD) simulations. Additionally, gene ontology (GO) and Kyoto Encyclopedia of Genes and Genomes (KEGG) enrichment analyses were conducted, highlighting the potential of these thunder god vine molecules for treating obesity. The molecular docking results from Discovery Studio indicated that these active components activate PPARG while inhibiting PTGS2.

## 2. Results and Discussion

### 2.1. Potential Targets for the Thunder God Vine and Obesity

We obtained 139 small molecules contained in the thunder god vine from online databases such as TCMSP [27] and TCMID [28], which serve as the foundation for studying their pharmacological activity and potential therapeutic effects. To gain a better understanding of the possible mechanisms of action of these small molecules, we queried the corresponding gene names of related target proteins through the UniProt database [29], aiming to establish a connection between the small molecules and their targets.

Additionally, we conducted an in-depth study of the genes associated with obesity. From the OMIM [30], DigSee [31], and GeneCards [32] databases, we retrieved 2429 pathogenic genes and targets related to obesity in humans. These genes play a crucial role in the onset and progression of obesity. Understanding their functions and interactions will aid in the identification of potential drug targets and provide new insights for obesity treatment.

By comparing the small molecules from the thunder god vine with these obesity-related targets, we can further explore the potential of the thunder god vine in anti-obesity applications. This process lays the groundwork for a subsequent network pharmacology analysis, enabling us to comprehensively assess the pharmacological properties of the thunder god vine and its contributions to obesity treatment.

### 2.2. Cluster of Analysis

First, we introduced the TriDimensional Hierarchical Fingerprint Clustering with Tanimoto Representative Selection (3DHFC-TRS) clustering algorithm to perform a clustering analysis of the 139 known molecules in the thunder god vine.

Clustering compounds based on molecular fingerprint similarity is a crucial method in drug discovery and virtual screening [33,34]. In this study, we implemented a clustering algorithm known as TriDimensional Hierarchical Fingerprint Clustering with Tanimoto Representative Selection (3DHFC-TRS) using R programming. Initially, we computed the MACCS molecular fingerprints to obtain a 2048-bit vector. We then utilized t-SNE for dimensionality reduction, projecting the fingerprints into three-dimensional space. This was followed by hierarchical clustering and the calculation of a similarity matrix using the Tanimoto coefficient [35]. Finally, we selected representative compounds from each cluster. In this research, we identified and compiled 139 small molecules extracted from the thunder god vine through a PubChem search.

**Figure 2:**
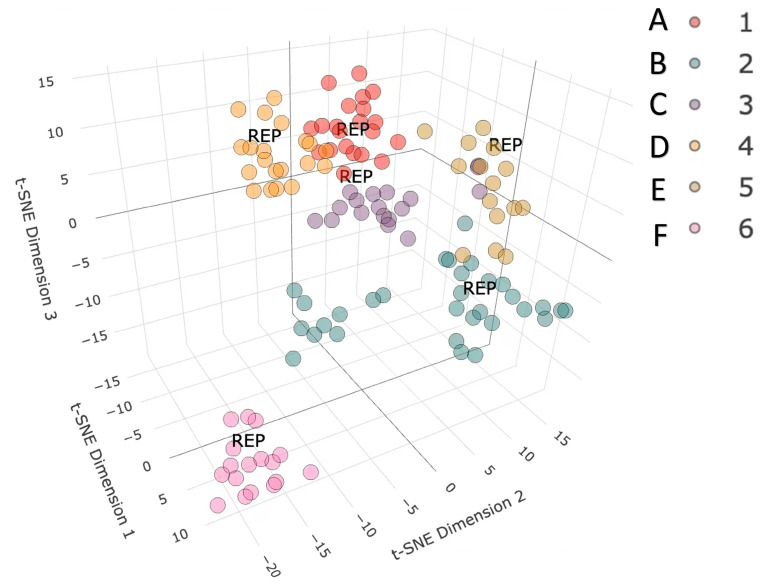
Cluster analysis of 139 molecules of the thunder god vine.(A) The first cluster, highlighted in red. (B) The second cluster, highlighted in cyan. (C) The third cluster, highlighted in purple. (D) The fourth cluster, highlighted in orange. (E) The fifth cluster, highlighted in yellow. (F) The sixth cluster, highlighted in pink.

In this study, a clustering analysis categorized the small molecules from the thunder god vine into six distinct classes, as illustrated in Figure 2. The first cluster, highlighted in red, is characterized by tripterygone as its representative molecule. The second cluster is marked in cyan, with Wilfotrine as its significant representative. The third cluster, shown in purple, identifies DBP as the representative molecule. The fourth cluster, distinguished in orange, features triptolide as its representative compound. The fifth cluster is highlighted in yellow, with Tripteroside defined as its key molecule. Lastly, the sixth cluster, represented in pink, includes Wilforlide B as its representative compound.

### 2.3. Network Pharmacology Results

Through a clustering analysis, we selected six representative molecules from the various clusters. A subsequent network pharmacology analysis revealed that the representative small molecule targets from the first cluster are closely associated with obesity targets. Using a Venn diagram [36], we identified the top six small molecules in the first cluster based on the number of intersecting genes. In our analysis of the intersections between the representative molecules of each group and their predicted targets related to obesity, we specifically focused on the performance of tripterygone, the representative small molecule from the first cluster.

**Figure 3:**
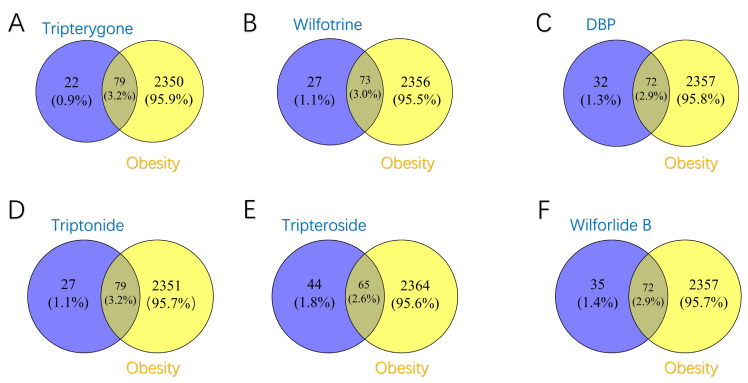
The intersection of the predicted targets and obesity targets of the representative six groups of small molecules. (**A**) Tripterygone, (**B**) Wilfotrine, (**C**) DBP, (**D**) Triptonide, (**E**) Tripteroside, and (**F**) Wilforlide B.

As shown in Figure 3A–F, there is a significant overlap between the predicted targets of tripterygone and obesity-related target genes, particularly notable genes such as AR, RARG, and HMGCR, which display important associations. These genes play critical roles in fat metabolism and the mechanisms underlying obesity. Given that these target genes are central to obesity-related pathways, the high overlap between the predicted targets and the obesity-related genes suggests the potential of tripterygone and its related molecules for obesity treatment.

Building on this finding, we turned our attention to Celastrol, a natural compound derived from the thunder god vine that has garnered widespread recognition for its potential in obesity treatment [37]. Given the strong link between Celastrol and obesity therapy, we specifically focused on the first cluster of molecules that share similar targets or pathways. We conducted a thorough analysis of these molecules to evaluate their potential as candidate drugs for obesity treatment.

To identify the small molecules with high potential in the first cluster, we ranked them based on the number of intersections with obesity-related target genes, as shown in Figures S1–S3. The results indicated that the top six small molecules exhibit significant overlap with obesity-related targets, suggesting potential therapeutic efficacy. This ranking allows us to select the most promising small molecules for further investigation, facilitating subsequent functional experimental validation.

**Figure 4:**
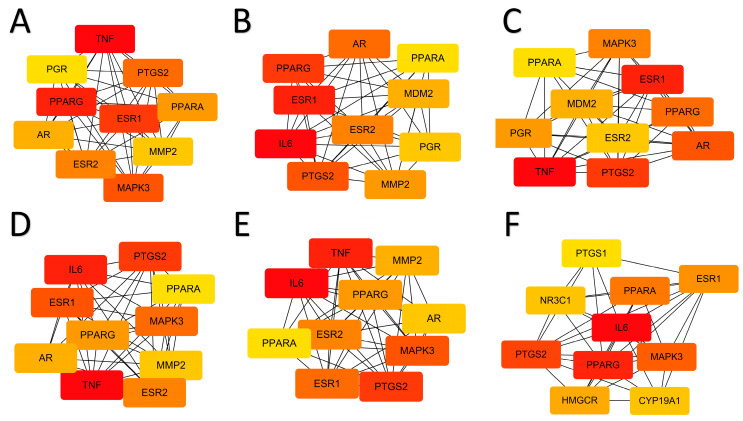
Using the Cytoscape analysis tool, we selected six molecules that correspond to the top ten hub genes based on their degree. (**A**) 3-Epikatonic Acid, (**B**) Hederagenin, (**C**) Triptonide, (**D**) Triptotriterpenic Acid B, (**E**) Triptotriterpenic Acid C, and (**F**) Ursolic Acid.

To gain a more systematic understanding of the interactions between these small molecules and obesity-related target genes, we constructed a protein–protein interaction (PPI) network using Cytoscape 3.8.0 software [38], as shown in Figures S4–S9. This network allows us to visually observe the roles of these small molecule target genes within obesity-related molecular pathways. An analysis revealed several key hub genes within the PPI network that are characterized by high connectivity and central regulatory roles. Utilizing the analysis tools in Cytoscape 3.8.0, we identified the top ten hub genes, among which PTGS2 and PPARG stand out prominently, Figure 4A–F, as they occupy central positions in the network and exhibit significant regulatory capacity.

PTGS2 is intricately linked to inflammatory responses, while PPARG serves as a critical regulatory factor in adipocyte differentiation and lipid metabolism. PTGS2 plays a significant role in the chronic inflammation associated with obesity, which contributes notably to obesity-related complications. Conversely, PPARG functions as a central transcription factor in adipogenesis and lipid metabolism, which is closely associated with obesity and its metabolic outcomes. Therefore, these genes are crucial to the onset and progression of obesity.

The topological analysis of the PPI network identified PTGS2 and PPARG as central hubs, reinforcing their crucial roles in obesity-related molecular pathways. Figures S4–S9 clearly illustrates the positions of these hub genes within the network and their interactions with other molecules. These findings provide a strong theoretical foundation for further investigating the potential therapeutic effects of small molecules on obesity, as well as offering new research directions for developing obesity treatment drugs.

As shown in Figures S10–S15, enrichment analysis of the gene ontology (GO) and Kyoto Encyclopedia of Genes and Genomes (KEGG) pathways revealed that the primary biological processes associated with these six small molecules include the regulation of inflammatory response, cellular ketone metabolism, steroid metabolism, negative regulation of lipid storage, and lipid localization. Additionally, the enriched cellular components include membrane rafts, membrane microdomains, lipid droplets, neuronal cell bodies, and synaptic membranes.

Further analysis indicated that the GO-identified molecular functions include nuclear receptor activity, ligand-activated transcription factor activity, and steroid binding. Meanwhile, KEGG analysis highlighted pathways associated with core targets, such as the PPAR signaling pathway and the insulin resistance pathway. These results suggest that the enriched pathways of thunder god vine target proteins are closely related to obesity and associated inflammation.

Next, we will conduct molecular docking of the six small molecules from the first cluster of the thunder god vine with PPARG and PTGS2 to further explore their interactions and potential therapeutic mechanisms.

### 2.4. Batch Molecular Docking and Machine-Learning Prediction Results for the First Cluster of Molecular from the Thunder God Vine Discs with PPARG and PTGS2

Table 1 presents the binding energies of the six representative compounds from Cluster 1 with the targets PPARG and PTGS2.

The molecular docking results shown in Table 1 indicate that the six representative compounds from the thunder god vine, namely ursolic acid, hederagenin, Triptotriterpenic acid B, Triptotriterpenic acid C, 3-epikatonic acid, and Triptonide exhibit a stronger binding affinity with PTGS2, while also demonstrating notable affinity with PPARG. This suggests that the interactions between these compounds and the receptors are relatively stable.

Discovery Studio also performed molecular docking of the two primary target proteins with the six active compounds, revealing that they activate PPARG [39] and inhibit PTGS2 [40].

The docking energies of 3-Epikatonic Acid, Hederagenin, Triptonide, Triptotriterpenic Acid B, Triptotriterpenic Acid C, and Ursolic Acid with PPARG are −2.3, −0.6, −6.5, 4.4, −3.1, and −2.8 kcal/mol, respectively. The docking energies with PTGS2 are −8.7, −8.8, −8.5, −8.5, −8.6, and −8.5 kcal/mol, respectively.

The molecular docking results shown in Table 1 indicate that Ursolic Acid, Hederagenin, Triptotriterpenic Acid B, Triptotriterpenic Acid C, 3-Epikatonic Acid, and Triptonide, the six representative molecules from the thunder god vine, exhibit a stronger binding affinity with PTGS2, while also demonstrating considerable affinity for PPARG. This suggests that the interactions of these six molecules with their respective receptors are relatively stable.

**Figure 5:**
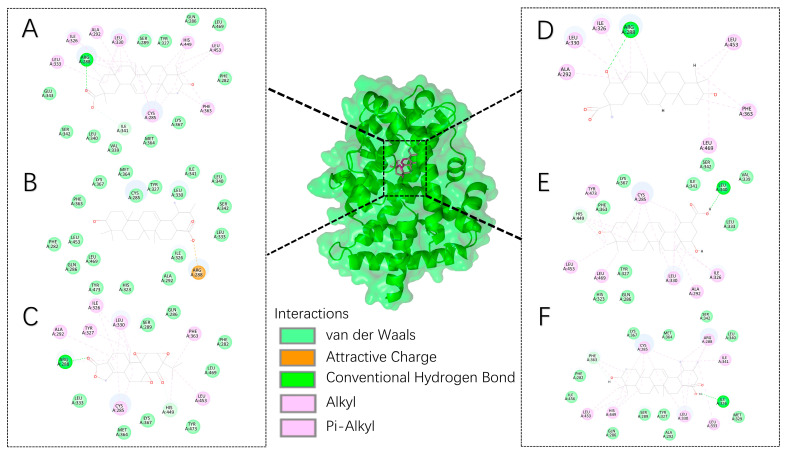
Visualization of molecular docking of six compounds with PPARG. (**A**) 3-Epikatonic Acid, (**B**) Hederagenin, (**C**) Triptonide, (**D**) Triptotriterpenic Acid B, (**E**) Triptotriterpenic Acid C, and (**F**) Ursolic Acid.

The molecules involved in the molecular docking have been visualized, with the binding sites to PPARG displayed in the corresponding figure, Figure 5A–F. The binding interactions between 3-Epikatonic Acid and PPARG include a conventional hydrogen bond with R288 and alkyl or Pi-alkyl interactions with C285, F363, L453, H449, L330, A292, I326, and L333. For Hederagenin, the primary binding interaction with PPARG is a salt bridge formed with R288. The main interactions of Triptonide with PPARG involve a conventional hydrogen bond with R288, along with alkyl or Pi-alkyl interactions with C285, L453, L330, F363, I326, Y327, and A292. The key interactions of Triptotriterpenic Acid B with PPARG also feature a conventional hydrogen bond with R288, in addition to alkyl or Pi-alkyl interactions with L453, F363, L469, L330, A292, and I326. For Triptotriterpenic Acid C, the primary interaction with PPARG is a conventional hydrogen bond with L340, as well as alkyl or Pi-alkyl interactions with L330, A292, I326, C285, Y473, L453, and L469. Lastly, the main interactions of Ursolic Acid with PPARG include a conventional hydrogen bond with I326 and alkyl or Pi-alkyl interactions with C285, I341, R288, L453, H449, L330, and L333.

**Figure 6:**
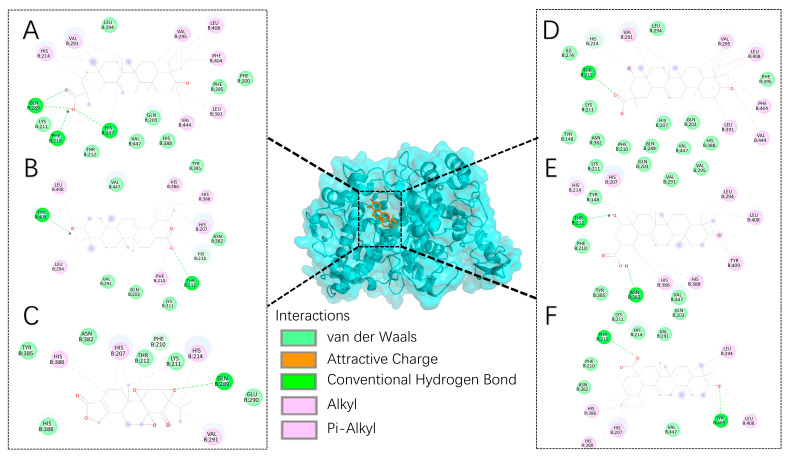
Visualization of molecular docking of six compounds with PTGS2 (**A**) 3-Epikatonic Acid, (**B**) Hederagenin, (**C**) Triptonide, (**D**) Triptotriterpenic Acid B, (**E**) Triptotriterpenic Acid C, and (**F**) Ursolic Acid.

The binding sites of the docked molecules with PTGS2 are shown in Figure 6A–F. The primary interactions of 3-Epikatonic Acid with PTGS2 involve forming conventional hydrogen bonds with the amino acid residues Q289, F210, and H207, as well as alkyl or Pi-alkyl interactions with V444, L391, F404, L408, V295, V291, and H214. For Hederagenin, the main interactions with PTGS2 include conventional hydrogen bonds with T212 and Y409, and alkyl or Pi-alkyl interactions with F210, H207, H388, H386, L408, and L294. The primary interactions of Triptonide with PTGS2 involve a conventional hydrogen bond with Q289 and alkyl or Pi-alkyl interactions with V291, H214, H207, and H386. For Triptotriterpenic Acid B, the main interactions with PTGS2 include a conventional hydrogen bond with T212 and alkyl or Pi-alkyl interactions with V444, L391, F404, L408, V295, and V291. The primary interactions of Triptotriterpenic Acid C with PTGS2 consist of conventional hydrogen bonds with both T212 and N382, along with alkyl or Pi-alkyl interactions with H386, H388, Y409, L408, L294, H207, and H214. Lastly, Ursolic Acid interacts with PTGS2 primarily through conventional hydrogen bonds with Y409 and T212, along with alkyl or Pi-alkyl interactions with L408, L294, H386, H388, and H207.

### 2.5. Results of Molecular Dynamics Simulations

#### 2.5.1. Molecular Dynamics Properties of the Twelve Systems

**Figure 7:**
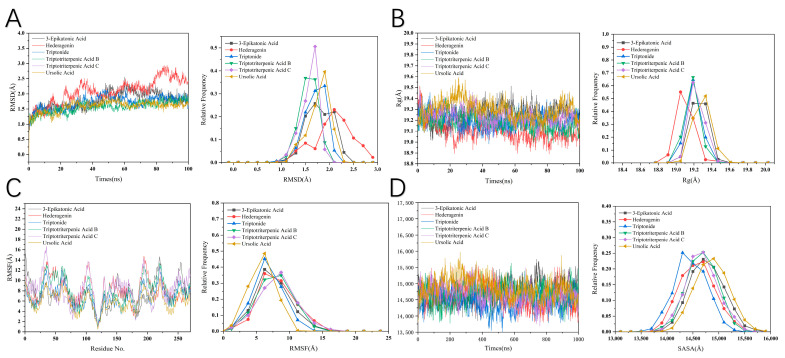
Structural stability analysis. (3-Epikatonic Acid, highlighted in black. Hederagenin, highlighted in red. Triptonide, highlighted in blue. Triptotriterpenic Acid B, highlighted in green. Triptotriterpenic Acid C, highlighted in purple. Ursolic Acid, highlighted in yellow.) (**A**) Temporal evolution of the RMSD from their initial structures for four systems. (**B**) Radius of gyration of four systems over a 100 ns MD simulation. (**C**) Root-mean-square fluctuation (RMSF) of the four systems. (**D**) SASA during a 100 ns MD simulation.

The stability of the six PPARG systems was assessed by calculating the root-mean-square deviation (RMSD) of the CA atoms, as shown in Figure 7A. All six MD trajectories reached equilibrium after approximately 50 ns, with all systems gradually maintaining stability. Notably, after overall equilibrium was achieved. The RMSD value for the Hederagenin–PPARG system was higher than that of the other systems, indicating stronger stability and minimal structural variation. Furthermore, the RMSD values for all six systems were below 3 Å, suggesting that there were no significant conformational changes.

In comparison to the other PPARG protein systems, the RMSD of the Hederagenin–PPARG system highlights the impact of activated conformations. As illustrated in Figure 7B, the radius of gyration (R_g_) of the Hederagenin–PPARG system exhibited the lowest value after 20 ns, indicating that its molecules are more compact. This suggests that the molecular assembly in the Hederagenin–PPARG system is denser, forming a more stable structure with better conformational stability compared to the other systems.

The solvent-accessible surface area (SASA) was utilized to estimate the number of residues exposed on the protein surface versus those buried within the hydrophobic core. As shown in Figure 7D, the SASA value for the Hederagenin–PPARG system exhibited minimal fluctuations during the simulation process and remained the lowest after 60 ns. This observation indicates that the molecular surface area in the Hederagenin–PPARG system is relatively small, supporting the conclusion of its compact molecular structure. The results for the SASA values are consistent with the R_g_ findings, further confirming the structural stability and compactness of this system.

**Figure 8:**
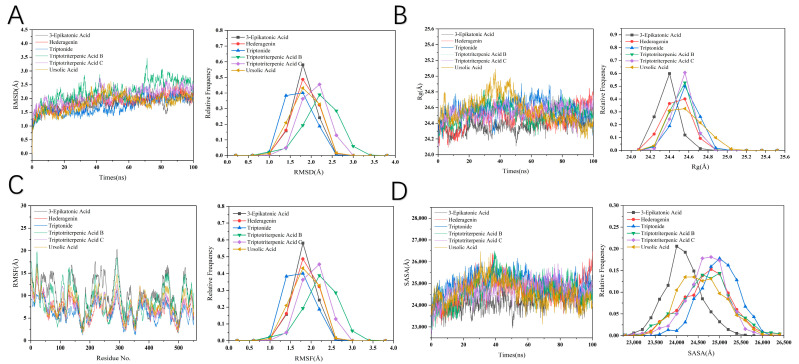
Structural stability analysis. (3-Epikatonic Acid, highlighted in black. Hederagenin, highlighted in red. Triptonide, highlighted in blue. Triptotriterpenic Acid B, highlighted in green. Triptotriterpenic Acid C, highlighted in purple. Ursolic Acid, highlighted in yellow.) (**A**) RMSD from their initial structures for four systems. (**B**) Radius of gyration of four systems over a 100 ns MD simulation. (**C**) RMSF of the four systems. (**D**) SASA during a 100 ns MD simulation.

Similarly, the stability of the six PTGS2 systems was evaluated by calculating the root-mean-square deviation (RMSD) of the CA atoms, as illustrated in Figure 8A. All six MD trajectories reached equilibrium after approximately 70 ns, with all systems gradually maintaining stability. Notably, after achieving overall equilibrium, the RMSD value for the Triptotriterpenic Acid B–PTGS2 system was higher than that of the other systems, indicating stronger stability and minimal structural variation. Additionally, the RMSD values for the five systems, excluding Triptotriterpenic Acid B–PTGS2, were all below 3 Å, suggesting that these systems did not undergo significant conformational changes. The RMSD of the Triptotriterpenic Acid B–PTGS2 system underscores the impact of the inhibitory conformation.

However, as shown in Figure 8A,B, the radius of gyration (R_g_) of the Triptotriterpenic Acid B-PTGS2 system reached its lowest value around 60 ns, but did not maintain this level. Moreover, the R_g_ values for all six systems were higher than those of the PPARG systems, indicating that the molecular structure of the PTGS2 systems is less stable compared to the PPARG systems.

The SASA was utilized to estimate the number of residues exposed on the protein surface versus those buried within the hydrophobic core. As depicted in Figure 8D, the SASA value for the Triptotriterpenic Acid C–PTGS2 system exhibited minimal fluctuations during the simulation but did not consistently maintain the lowest value. This observation suggests that the molecular surface area in the PPARG systems is smaller than that in the PTGS2 systems, supporting the conclusion that the molecules in the PPARG systems are more compact. The results for the SASA values align with the R_g_ findings, further confirming the structural stability and compactness of the PPARG system compared to the PTGS2 system.

#### 2.5.2. Analysis of the Interaction Between Protein and Inhibitors

To more accurately assess the energy differences and avoid the misleading evaluations associated with rigid receptor docking studies, we performed 100 ns molecular dynamics simulations on these six compounds, followed by an MM/PBSA energy analysis. As shown in Table 2 and Table 3, the binding of the molecules to PPARG demonstrates stronger binding energy, with Hederagenin exhibiting the highest binding affinity for PPARG.

Therefore, we can conclude that the Hederagenin–PPARG system exhibits the highest structural stability, which is consistent with the results obtained from the MM/PBSA analysis.

In this 100 ns simulation with 1000 frames, we selected the three small molecules with the lowest binding energies to PPARG, namely Hederagenin, Triptotriterpenic Acid B, and Triptotriterpenic Acid C, for a hydrogen bond analysis, as shown in Table S1. During the simulations of these three systems, the average numbers of hydrogen bonds per frame were 2.1, 0.41, and 1.42, respectively. We noted that the Triptotriterpenic Acid B system formed fewer hydrogen bonds during the MD simulation, yet it had a lower binding energy. This suggests that the dominant forces within this system are not hydrogen bonds but possibly van der Waals forces, as illustrated in Figure S17. In contrast, hydrogen bonds were the primary interactions in the Hederagenin–PPARG and Triptotriterpenic Acid C–PPARG systems. According to Table S3, in the Hederagenin–PPARG system, hydrogen bonds predominantly formed between the donor ARG81-Side and the acceptor 6Q5271-Side, with a probability of 92.50%, as shown in Figure S16. For the Triptotriterpenic Acid C–PPARG system, hydrogen bonds mainly formed between the donor UNK271-Side and the acceptor GLU136-Side, with a probability of 67.20%, as depicted in Figure S18.

In this 100 ns simulation with 1000 frames, we selected the three small molecules with the lowest binding energies to PTGS2, namely Ursolic Acid, Hederagenin, and Triptotriterpenic Acid B, for hydrogen bond analysis, as shown in Table S2. During the simulations of these three systems, the average numbers of hydrogen bonds per frame were 0.38, 0.67, and 0.95, respectively. We noticed that the Hederagenin–PTGS2 and Ursolic Acid–PTGS2 systems formed fewer hydrogen bonds during the MD simulation, despite having lower binding energies. This suggests that the dominant forces within these two systems are not hydrogen bonds but likely van der Waals forces, as illustrated in Figures S19 and S20. In contrast, in the Triptotriterpenic Acid B–PTGS2 system, hydrogen bonds were the primary interaction. According to Table S4, in the Triptotriterpenic Acid B–PTGS2 system, hydrogen bonds mainly formed between the donor UNK552-Side and the acceptor ALA168-Main, with a probability of 59.30%, as shown in Figure S21.

### 2.6. Functional Group Analysis

**Figure 9:**
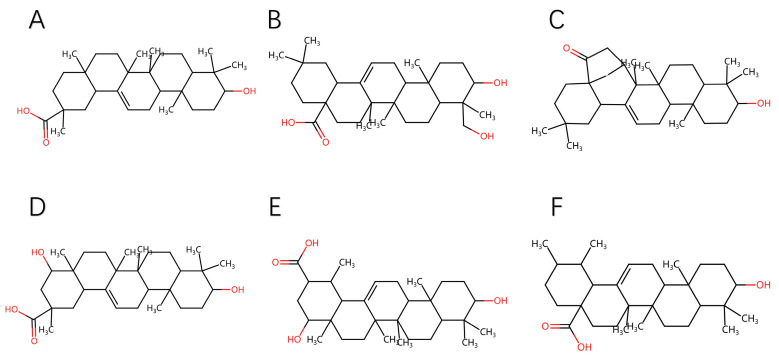
Planar structures of the six molecules, the red parts indicate the presence of oxygen atoms. (**A**) 3-Epikatonic Acid, (**B**) Hederagenin, (**C**) Triptonide, (**D**) Triptotriterpenic Acid B, (**E**) Triptotriterpenic Acid C, and (**F**) Ursolic Acid.

The Hederagenin–PPARG system demonstrates exceptional structural stability, likely due to the unique pentacyclic triterpene backbone of the Hederagenin molecule, as illustrated in Figure 9A–F. Pentacyclic triterpenes possess a rigid and highly stable chemical framework that provides a solid foundation for molecular binding. Notably, the hydroxyl group at the C3 position, along with the hydroxyl and carboxyl groups near C5, may play a crucial role in its interaction with the PPARG receptor.

These functional groups may enhance the binding of Hederagenin to the active site of PPARG through non-covalent interactions, such as hydrogen bonding. The presence of these hydroxyl and carboxyl groups not only provides additional polarity to strengthen inter-molecular affinity but also contributes to stabilizing the molecular conformation, reducing fluctuations in free energy, and ultimately improving the overall stability of the system. Compared to other triterpene compounds, these distinctive structural features of Hederagenin may explain its stronger binding capacity and structural stability within the PPARG system.

Therefore, we hypothesize that the rational spatial distribution of these key functional groups, in conjunction with the triterpene backbone, allows Hederagenin to exhibit higher binding affinity and stability in the PPARG system. This finding further supports the potential of Hederagenin as a PPARG modulator and may provide new insights for developing PPARG-targeted drugs based on triterpene compounds.

### 2.7. Functional Association Network Analysis of the Two Hub Targets Was Conducted Using GeneMANIA

**Figure 10:**
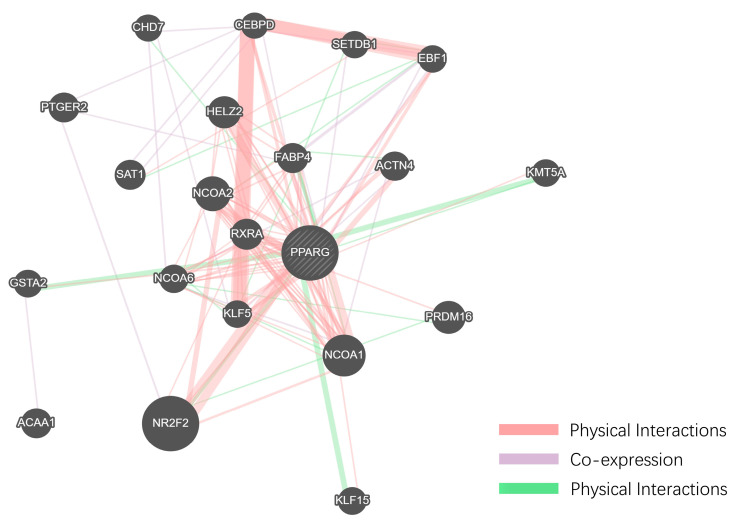
Screening of additional genes associated with PPARG in the GeneMANIA framework.

**Figure 11:**
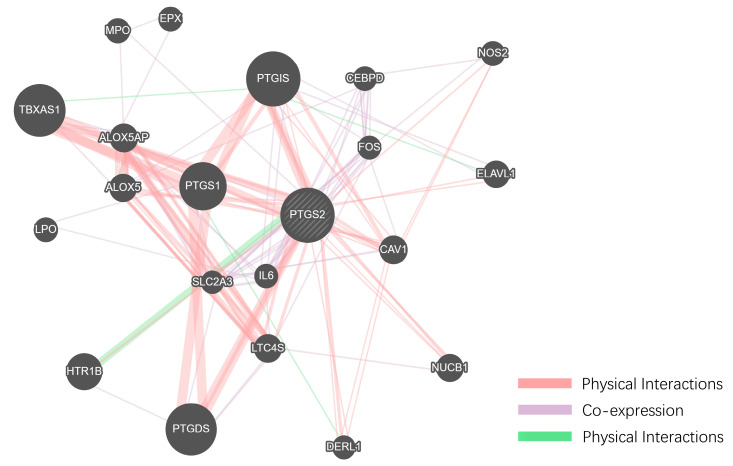
Screening of additional genes associated with PTGS2 in the GeneMANIA framework.

To comprehensively identify the functional genes associated with the two hub targets of the thunder god vine molecules relevant to obesity, we introduced an innovative integration approach within the GeneMANIA framework. As shown in Figure 10 and Figure 11, 20 additional related genes were screened for each hub target, expanding the gene lists of the two key targets, PPARG and PTGS2, to construct the GMFA Expanded Database (GMFA-ED). This process not only deepened our understanding of the mechanisms of action of thunder god vine molecules but also broadened and enriched the gene network’s scope and depth.

After removing the duplicate genes, the database comprises a total of 41 genes, establishing a foundation for further in-depth analysis of gene interactions. The GMFA method integrates co-expression, genetic interaction, physical interaction, and various other functional relationships, aiming to comprehensively capture the key genes closely linked to the pathological processes of obesity. This approach allows us to better understand the potential of thunder god vine molecules in the regulation of the biological pathways related to obesity and to identify potential therapeutic targets.

The GMFA-ED database not only enhances existing gene resources but also provides a reliable foundation and a more comprehensive genetic perspective for researching the application of thunder god vine molecules in obesity treatment. We anticipate that utilizing this database will facilitate the development of thunder god vine molecules for treating obesity and open up new avenues for the creation of innovative therapies.

## 3. Materials and Methods

### 3.1. Clustering of Molecules

First, we utilized the “rcdk” package to calculate the MACCS molecular fingerprints, resulting in a 2048-bit vector [41]. Next, we applied t-SNE for dimensionality reduction, projecting the molecular fingerprints into three-dimensional space. We then performed hierarchical clustering on these reduced dimensions to form distinct clusters. Within each cluster, we used the Tanimoto coefficient to calculate a similarity matrix, assessing the similarity between the molecules. The molecule with the highest similarity to all other molecules within the cluster was selected as the representative compound for that specific group.

### 3.2. Network Pharmacology Analysis

#### 3.2.1. Predictive Analysis of Interactions Between Potential Obesity Targets and Receptor Components of Thunder God Vine

In our study, we initially identified potential obesity-related target genes from three prominent databases, including OMIM, DigSee, and GeneCards. The search in the GeneCards database revealed 1386 potential targets with a score of over 5. Meanwhile, OMIM cataloged 859 potential target genes, and the DigSee database comprehensively listed 770 potential target genes. After meticulously comparing and eliminating duplicate genes from these sources, we compiled a comprehensive list of 2429 potential target genes for further investigation. To predict the potential interactions between the active components of the six representative molecules from the thunder god vine and the obesity-related target genes, we utilized tools and databases, such as UniProt, to gather the target genes of these six representative clusters. We then visualized the unique overlapping target genes of Ursolic Acid, Hederagenin, Triptotriterpenic Acid B, Triptotriterpenic Acid C, 3-Epikatonic Acid, and Triptonide using a Venn diagram, highlighting their interactions with obesity-related diseases.

#### 3.2.2. Precise Construction of Protein–Protein Interaction Networks

To further analyze the protein–protein interaction (PPI) network between the six representative molecules and the obesity targets, we utilized the well-known bioinformatics tool called the STRING database [42]. Subsequently, we employed Cytoscape 3.8.0.software [43] to analyze the topological characteristics of the PPI network and select the top ten hub genes using Cytoscape’s analytical tools.

#### 3.2.3. Comprehensive Enrichment Analysis of GO and KEGG Pathways

In this study, we extensively utilized a suite of R packages, including BiocManager and clusterProfiler, to perform enrichment analyses of gene ontology (GO) biological processes [44,45] and Kyoto Encyclopedia of Genes and Genomes (KEGG) pathways [46]. The aim of these analyses is to elucidate the relationships between genes and the genome, as well as to clarify the functions and interactions of the target genes within biological systems. Through these analyses, we extracted detailed GO information from the tissues, which was then vividly visualized using bar charts and bubble plots to facilitate a better understanding and interpretation of our research findings.

#### 3.2.4. Molecular Docking of Active Components from the First Cluster of Thunder God Vine with PPARG and PTGS2

We used the cocrystal structure of the PPARγ LBD and NFKBIB/IKBβ peptide (PDB ID: 9CK0) model [47], which consists of 276 residues. Molecular docking was subsequently conducted using AutoDock Vina 1.2.0 [48], targeting the MOA channel to stimulate substrate entry, as the GW1929 factor is the activation site for PPARγ, a docking box was set up at this position. The docking box dimensions were set to 9.75 Å × 12.75 Å × 11.25 Å, with center coordinates of x = −23.208, y = −20.157, and z = 9.698. Based on the results of the clustering analysis and network pharmacology, we performed molecular docking of the six representative small molecules from the first cluster along with their target proteins. The interactions within the docking structures were visualized using Discovery Studio 2024 Visualizer.

Similarly, we utilized the cyclooxygenase-2 (PDB ID: 3HS5) model [49], which consists of 591 residues. Next, molecular docking was conducted using AutoDock Vina 1.2.0 to target the MOA channel, which is the substrate binding site. Small molecules act as competitive inhibitors in this region of PTGS2 by competing with the substrate. The docking box dimensions were set to 27 Å × 39 Å × 14.5 Å, with center coordinates of x = 22.312, y = 37.254, and z = 24.877. Based on the results of the clustering analysis and network pharmacology, we performed molecular docking of the six representative small molecules from the first cluster with PPARG and PTGS2, screening out the combination with the lowest docking energy as the optimal molecule and target. The interactions within the docking structures were visualized using Discovery Studio 2024 Visualizer [50]. 

### 3.3. Molecular Dynamics Simulations

Using the docking results of Ursolic Acid, Hederagenin, Triptotriterpenic Acid B, Triptotriterpenic Acid C, 3-Epikatonic Acid, and Triptonide, we conducted a single molecular dynamics (MD) simulation [51] and employed the Modeler plugin of UCSF Chimera [52] to model the missing structures of all of the molecules. The FF19SB AMBER force field [53] was used for the system, and the PMEMD engine in AMBER 22 [54] was utilized for the MD simulations. An OPC water model [55] and periodic boundary conditions were implemented to mitigate the edge effects throughout the simulation. A 10 Å buffer solution was maintained between the solute surface and the simulation box boundaries, with sodium ions added to ensure charge neutrality. During the process, the SHAKE algorithm [56] constrained all hydrogen bond interactions, while the PME method addressed non-bonded electrostatic forces. The initial system minimization involved 1500 steps under an enzymatic inhibition potential of 10 kcal/mol, followed by an additional 1500 steps of unconstrained minimization using the conjugate gradient method. While keeping the volume constant, we gradually heated the solute from 0 K to 310 K by applying harmonic potential constraints and a collision frequency of 1 ps. At 310 K, a 500 ps equilibration phase occurred before the main 100 ns simulation. The pressure was maintained at 1 bar using the Berendsen barostat [57]. The conditions remained consistent during the NPT integration, employing random seeding, a pressure coupling constant of 2 ps, and temperature regulation via a Langevin thermostat with a collision frequency of 1 ps.

In the analysis of ligand binding affinity, 20 snapshots taken between 0 and 100 ns of the trajectory were selected for evaluation using the MM/PBSA method [58], and the interactions within the systems were analyzed [59,60].

### 3.4. GeneMANIA-Based Functional Association Network Analysis

To comprehensively identify the genes functionally related to the two hub targets of thunder god vine in obesity treatment, we employed a novel approach within the GeneMANIA framework [61]. This GMFA network analysis method involves discovering 20 additional genes for each central gene, prioritizing those most strongly associated with the gene–gene network. The analysis focuses on three key parameters, namely co-expression, genetic interaction, and physical interaction, significantly enhancing the accuracy of therapeutic target identification.

The co-expression analysis aids in selecting the targets involved in shared disease-related processes, while a genetic interaction analysis identifies potential targets with complex network dependencies. Physical interaction analysis helps pinpoint critical components of disease-related networks for targeted intervention. By integrating these parameters, we gained a more nuanced understanding of the gene functions, facilitating the identification of robust therapeutic targets that play multifaceted roles in the disease process.

Subsequently, we integrated all newly identified genes with the initially obtained two hub targets to establish an expanded database based on GMFA, referred to as the GMFA-ED. This integration resulted in a total of 41 unique genes in the expanded database, providing a more comprehensive foundation for identifying potential therapeutic targets of the thunder god vine against obesity.

## 4. Conclusions

In this study, we thoroughly investigated the potential of small molecules extracted from the thunder god vine (Tripterygium wilfordii) for obesity treatment, with a particular focus on their activating effects on PPARG and inhibitory effects on PTGS2. By employing advanced computational strategies and innovative clustering algorithms, we successfully isolated a subset of bioactive molecules, including 3-Epikatonic Acid, Hederagenin, Triptonide, Triptotriterpenic Acid B, Triptotriterpenic Acid C, and Ursolic Acid. These small molecules, due to their significant PPARG activation and PTGS2 inhibition, demonstrate novel application potential in obesity treatment.

In the molecular docking of these six compounds with PPARG and PTGS2, we discovered that the complex formed by Hederagenin with PPARG exhibited the best docking energy among the small molecules. We investigated the relationship between the molecular structure and functional properties of these six small molecules, particularly focusing on Hederagenin. This finding provides a solid foundation and theoretical basis for further exploring the therapeutic potential of this interaction. Additionally, we developed an expanded database (GMFA-ED) through GMFA network analysis, which integrates the target genes associated with the thunder god vine and obesity, resulting in a total of 41 unique genes. This comprehensive resource lays the groundwork for identifying potential therapeutic targets of the thunder god vine in combating obesity.

During the molecular docking of these six compounds with PPARG and PTGS2, we identified the small molecule with the most favorable docking energy, which was Hederagenin in a complex with PPARG. Additionally, we explored the relationship between the molecular structure of Hederagenin and its functional properties. This finding provides a solid basis and rationale for subsequent studies, including animal experiments, to further explore the therapeutic potential of this interaction.

Through the analysis of the mechanisms of action of these small molecules, we offer a novel perspective for obesity treatment, highlighting the potential for discovering effective components from natural drugs. Our research underscores the importance of exploring traditional medicinal resources, such as the thunder god vine, to identify and validate bioactive compounds that may contribute to innovative therapeutic strategies against obesity.

Additionally, our research underscores the critical role of computational methods in identifying and evaluating new drug candidates. By utilizing computational simulations, we were able to predict the binding affinity and biological activity of these small molecules with their target proteins, providing a significant theoretical foundation for subsequent experimental studies. This research not only offers new directions for developing novel obesity treatments but also provides scientific support for the modernization of traditional Chinese medicine applications.

## Figures and Tables

**Table 1:** Molecular docking energy results for the six small molecules with PPARG and PTGS2.

Molecule	PPARG	PTGS2
3-Epikatonic Acid	−2.3	−8.7
Hederagenin	−0.6	−8.8
Triptonide	−6.5	−8.5
Triptotriterpenic Acid B	4.4	−8.5
Triptotriterpenic Acid C	−3.1	−8.6
Ursolic Acid	−2.8	−8.5

**Table 2:** MM/PBSA results for six small molecules with PPARG.

System	ΔE_vdw_	ΔE_ele_	ΔG_solv_	ΔG_gas_	ΔG_total_
3-Epikatonic Acid	−52.94 ± 3.81	−12.39 ± 7.76	32.12 ± 6.57	−65.34 ± 7.98	−33.22 ± 4.32
Hederagenin	−54.88 ± 3.73	−32.45 ± 18.59	47.21 ± 12.81	−87.33 ± 18.17	−40.12 ± 7.75
Triptonide	−45.31 ± 2.38	−10.15 ± 3.80	35.10 ± 4.49	−55.47 ± 5.23	−20.36 ± 3.49
Triptotriterpenic Acid B	−60.37 ± 3.09	−11.69 ± 3.98	36.82 ± 4.77	−72.06 ± 4.64	−35.24 ± 4.03
Triptotriterpenic Acid C	−58.80 ± 3.05	−16.75 ± 4.77	38.59 ± 4.12	−75.55 ± 5.18	−36.96 ± 5.30
Ursolic Acid	−60.23 ± 2.79	−7.18 ± 1.75	32.38 ± 2.18	−67.41 ± 3.09	−35.03 ± 3.33

**Table 3:** MM/PBSA results for six small molecules with PTGS2.

System	ΔE_vdw_	ΔE_ele_	ΔG_solv_	ΔG_gas_	ΔG_total_
3-Epikatonic Acid	−46.81 ± 3.47	−9.16 ± 6.00	30.95 ± 5.37	−55.97 ± 6.81	−25.03 ± 4.00
Hederagenin	−43.96 ± 3.74	2.78 ± 11.80	13.51 ± 10.54	−41.18 ± 11.19	−27.67 ± 4.45
Triptonide	−25.70 ± 4.91	−5.77 ± 5.35	17.40 ± 7.44	−31.46 ± 8.27	−14.06 ± 2.46
Triptotriterpenic Acid B	−46.53 ± 2.86	−7.10 ± 5.04	25.90 ± 5.52	−53.62 ± 6.03	−27.72 ± 3.04
Triptotriterpenic Acid C	−46.62 ± 3.49	−17.23 ± 4.84	37.68 ± 3.69	−63.86 ± 5.72	−26.18 ± 5.57
Ursolic Acid	−48.58 ± 4.19	−4.13 ± 6.36	25.52 ± 8.20	−52.71 ± 9.37	−27.19 ± 3.28

## Data Availability

All data are available on request from the authors.

## Supplementary Materials

The following supporting information can be downloaded at https://www.mdpi.com/article/10.3390/ijms252312501/s1.
