# Supplementary material for: Unveiling the Anti-Obesity Potential of Thunder God Vine: Network Pharmacology and Computational Insights into Celastrol-like Molecules
Source: Int J Mol Sci. 2024 Nov 21;25(23):12501. doi: 10.3390/ijms252312501 (PMC11641352; doi:10.3390/ijms252312501)
Supplement: Supplementary file 1 [file ijms-25-12501-s001.zip › ijms-3322425-supplementary/Supplementary/Supplementary Figures.pdf]

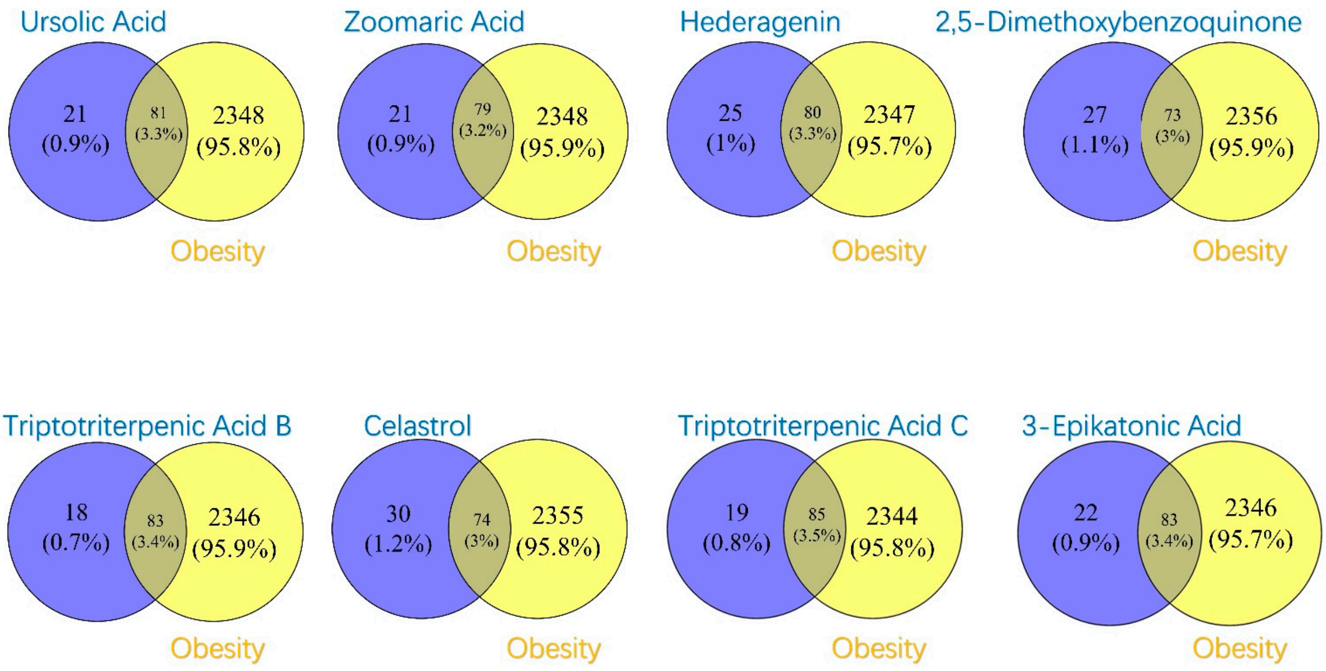

Figure S1. Intersection of Predicted Targets of Small Molecules with Obesity Targets

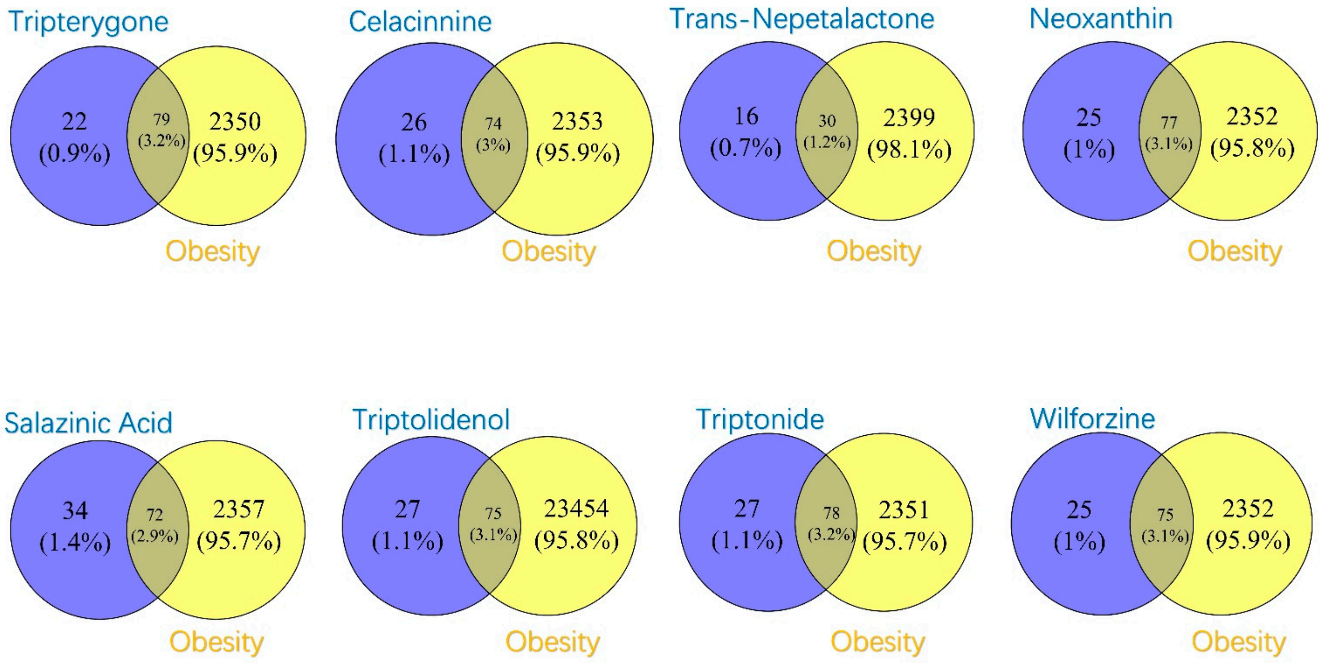

Figure S2. Intersection of Predicted Targets of Small Molecules with Obesity Targets

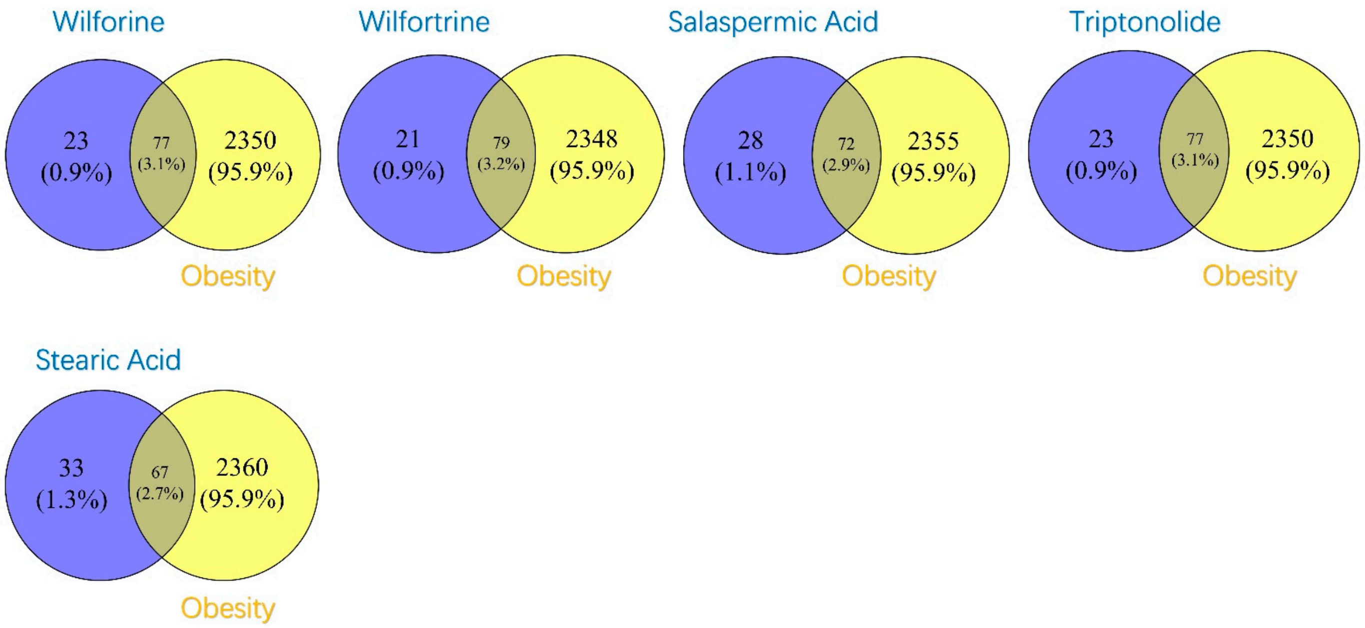

FigureS3 . Intersection of Predicted Targets of Small Molecules with Obesity Targe

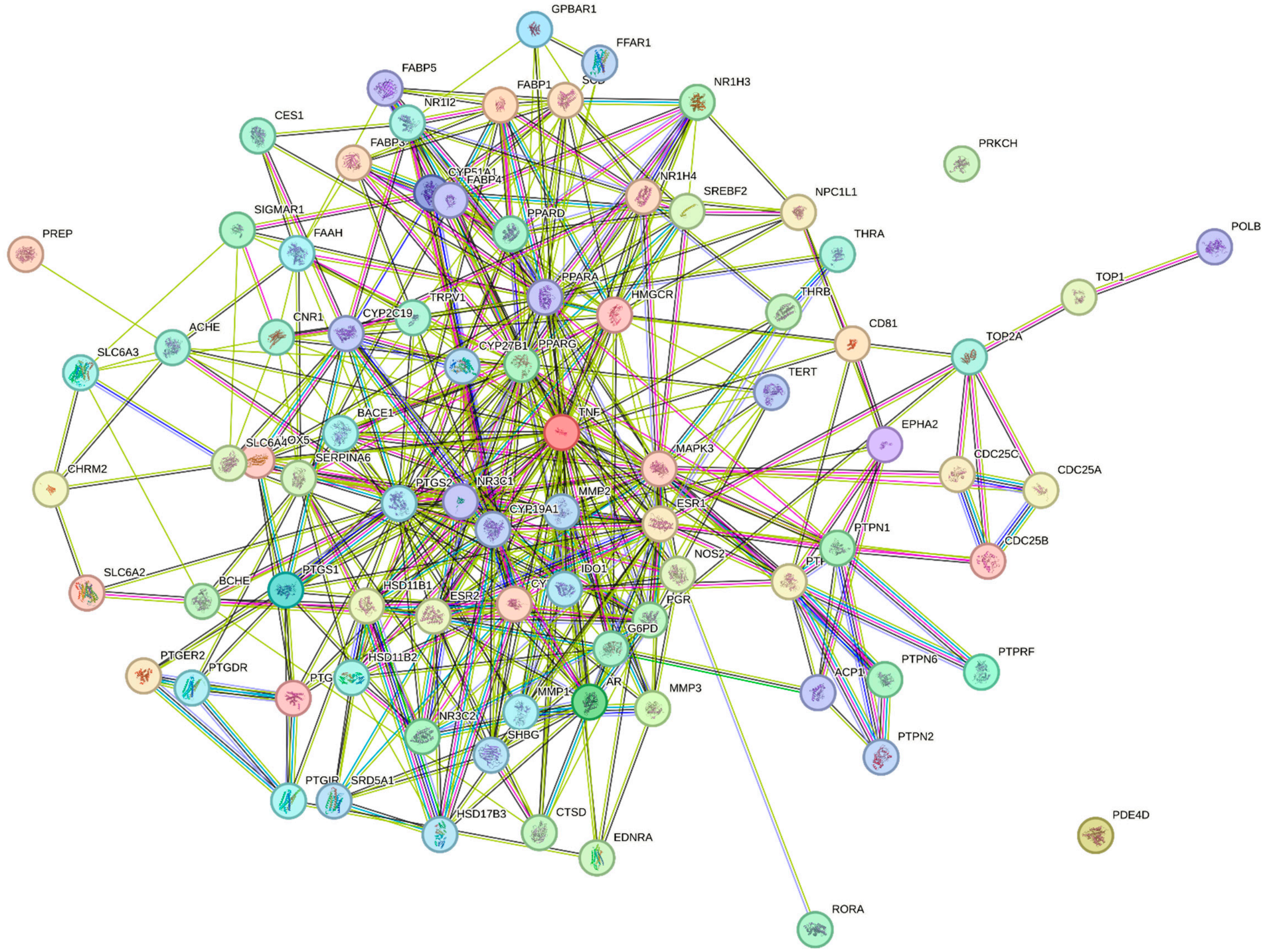

Figure S4. Analysis of Interactions between the 3-Epikatonic Acid and Obesity-Related Intersecting Genes Using

the STRING Database.

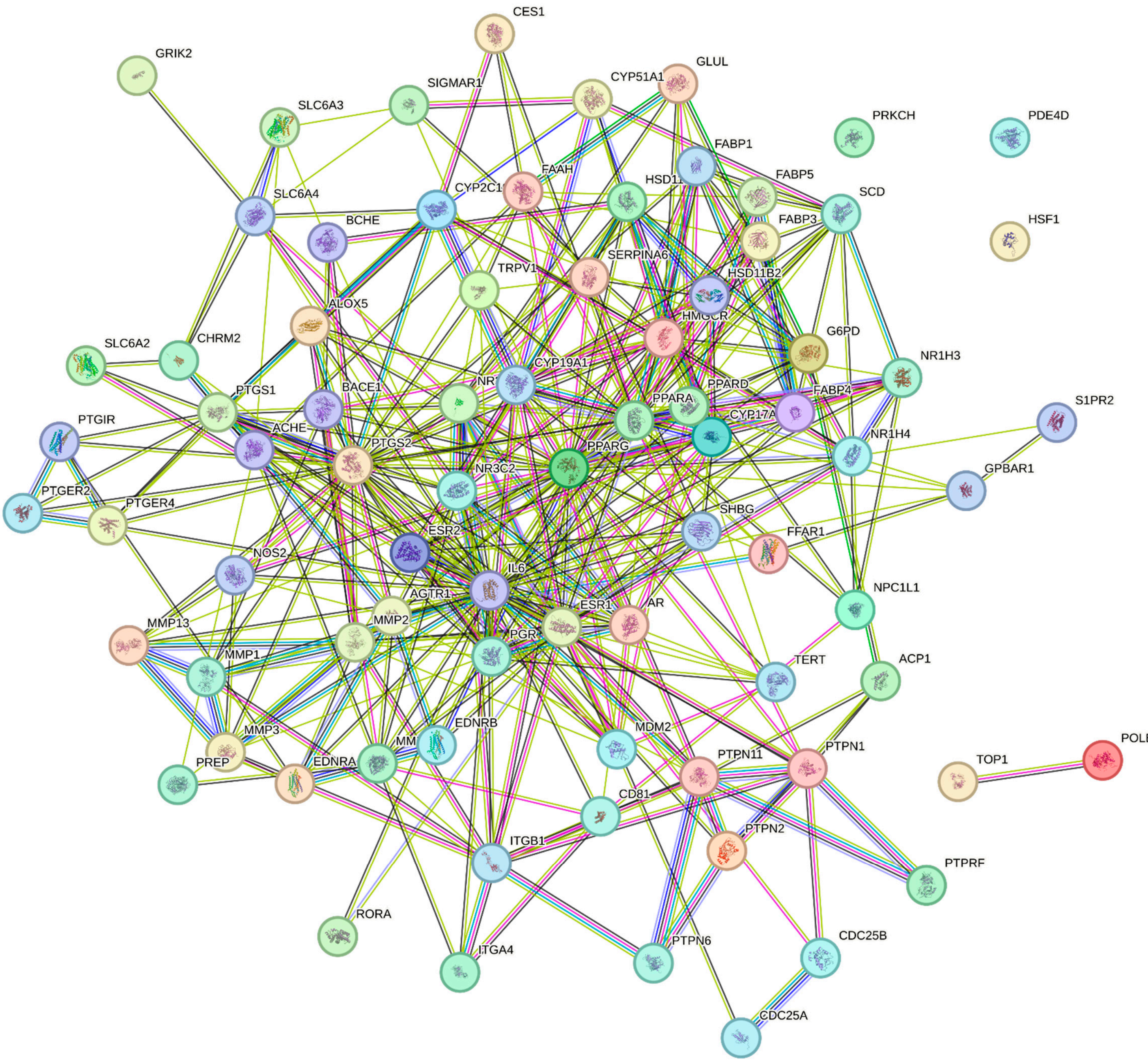

Figure S5. Analysis of Interactions between the Hederagenin and Obesity-Related Intersecting Genes Using the STRING Database.

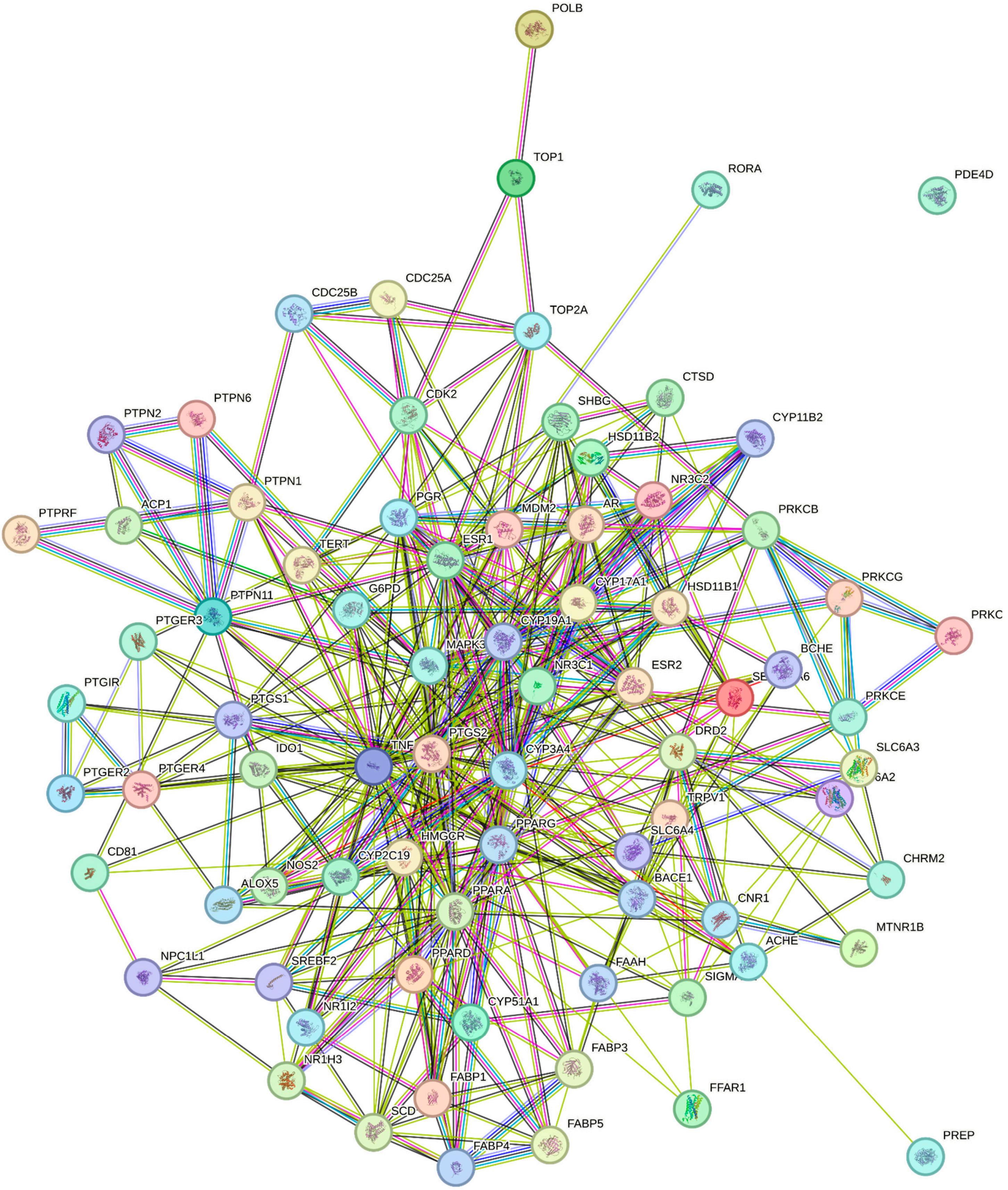

Figure S6. Analysis of Interactions between the Triptonide and Obesity-Related Intersecting Genes Using the STRING Database.

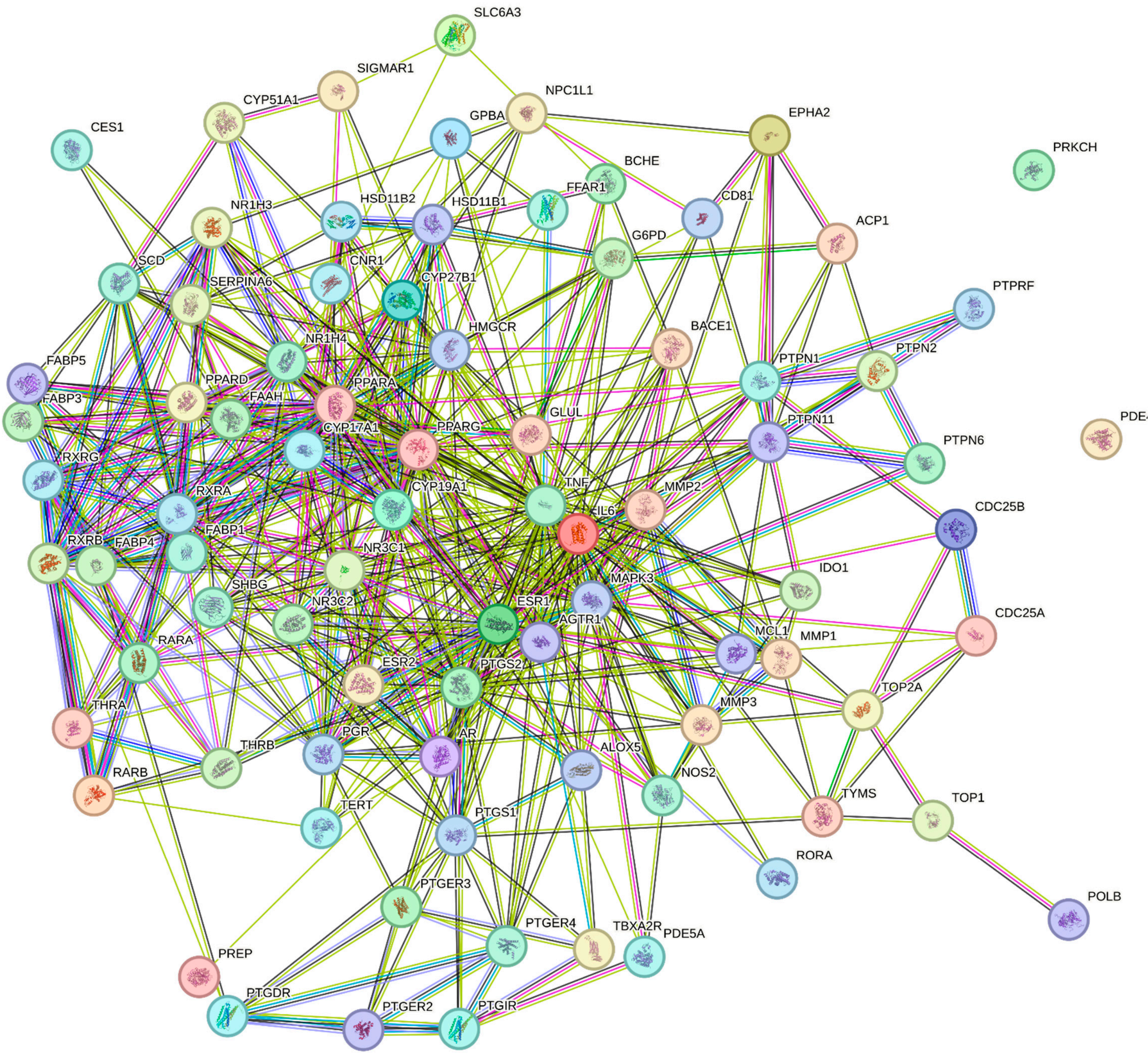

Figure S7. Analysis of Interactions between the Triptotriterpenic Acid B and Obesity-Related Intersecting Genes Using the STRING Database.

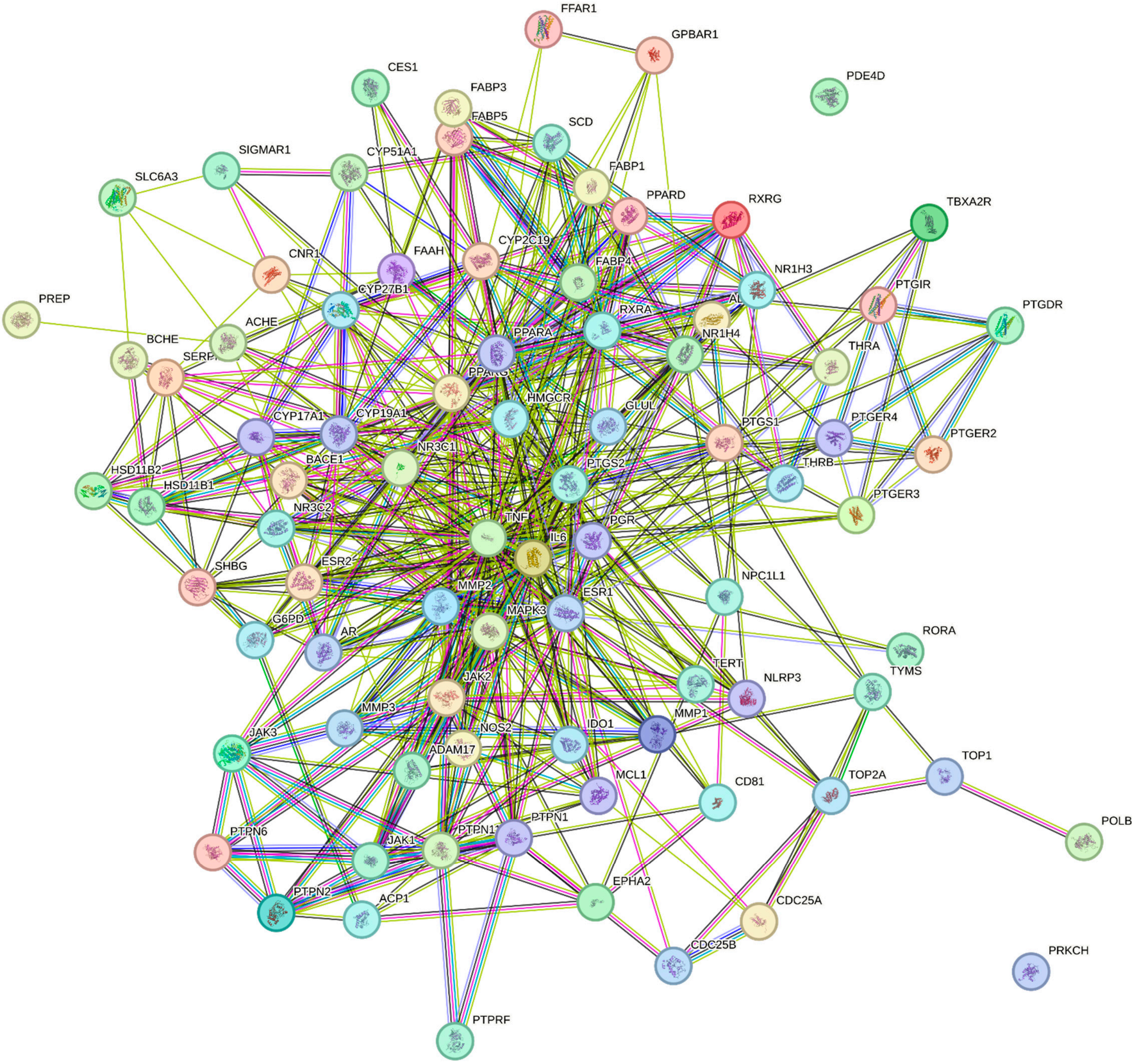

Figure S8. Analysis of Interactions between the Triptotriterpenic Acid C and Obesity-Related Intersecting Genes Using the STRING Database.

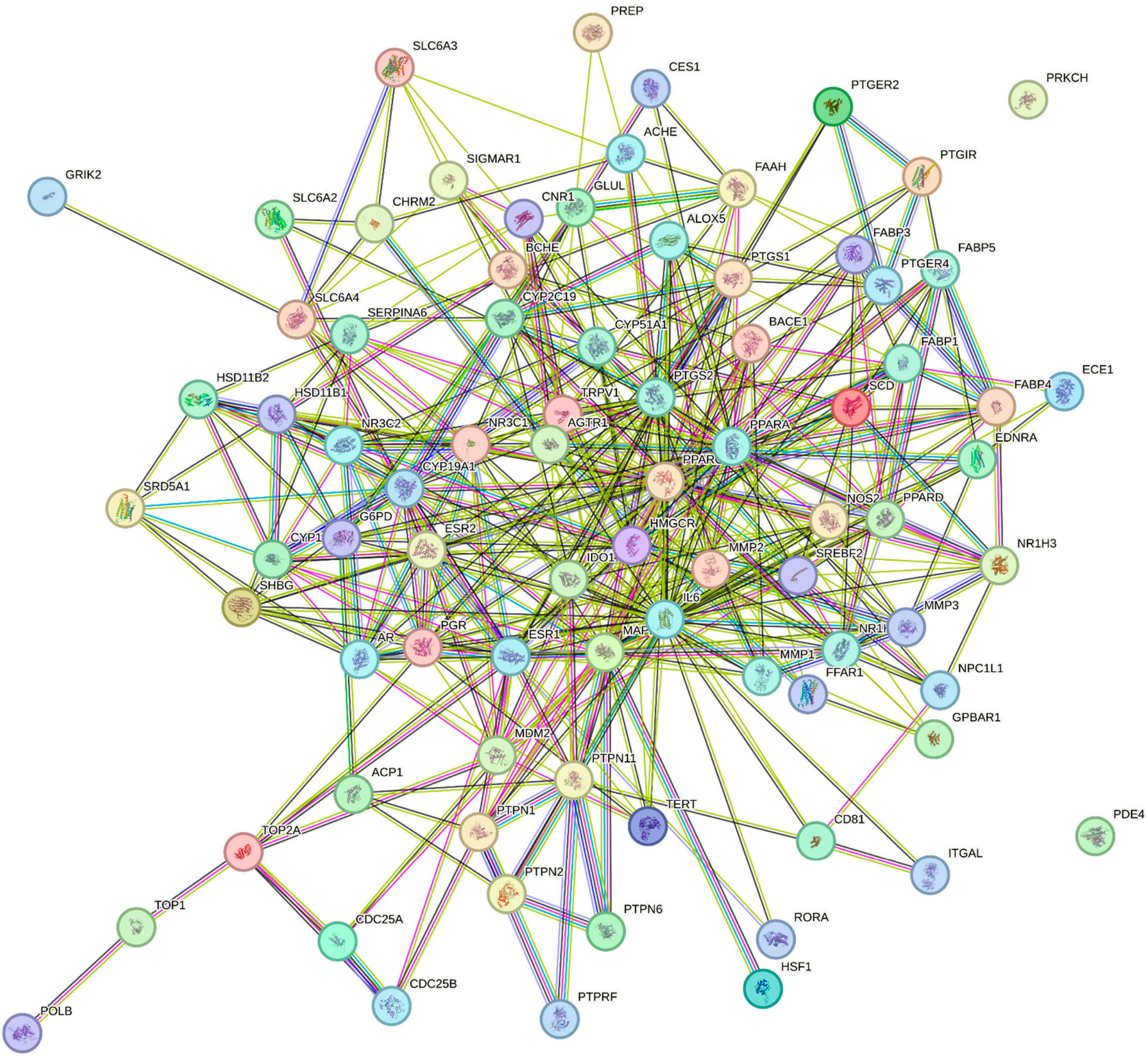

Figure S9. Analysis of Interactions between the Ursolic Acid and Obesity-Related Intersecting Genes Using the STRING Database.

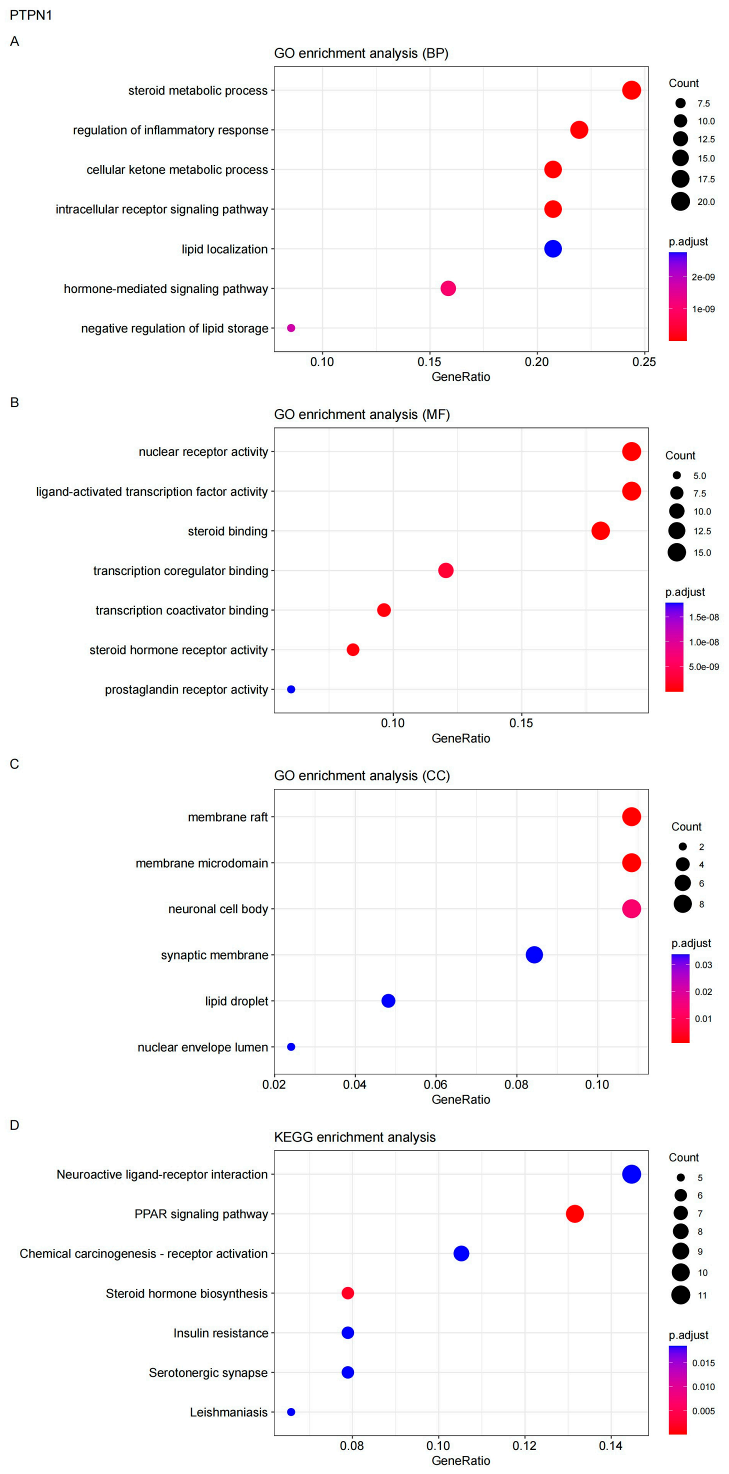

Figure S10.

The results of the GO and KEGG pathway enrichment analyses are visualized, with the size of the bubbles representing the number of genes. The GO analysis focuses on genes related to biological processes, molecular functions, and cellular components associated with 3-Epikatonic Acid and obesity. Meanwhile, the KEGG pathway enrichment analysis emphasizes the enriched pathways.

As shown in Figure S10 (A), the GO and KEGG pathway enrichment analyses revealed several potential biological processes associated with 3-Epikatonic Acid, including the steroid metabolic process, regulation of inflammatory response, cellular ketone metabolic process, intracellular receptor signaling pathway, lipid localization, hormone-mediated signaling pathway, and negative regulation of lipid storage.

Additionally, as shown in Figure S10 (B), the molecular functions identified through the GO analysis include nuclear receptor activity, ligand-activated transcription factor activity, steroid binding, transcription coregulator binding, transcription coactivator binding, steroid hormone receptor activity, and prostaglandin receptor activity.

As shown in Figure S10 (C), the enriched cellular components identified include membrane raft, membrane

microdomain, neuronal cell body, synaptic membrane, lipid droplet, and nuclear envelope lumen.

As shown in Figure S10 (D), the KEGG analysis identified enriched pathways containing the most critical targets, such as Neuroactive ligand-receptor interaction, PPAR signaling pathway, Chemical carcinogenesis-receptor activation, Steroid hormone biosynthesis, Insulin resistance, Serotonergic synapse, and Leishmaniasis.

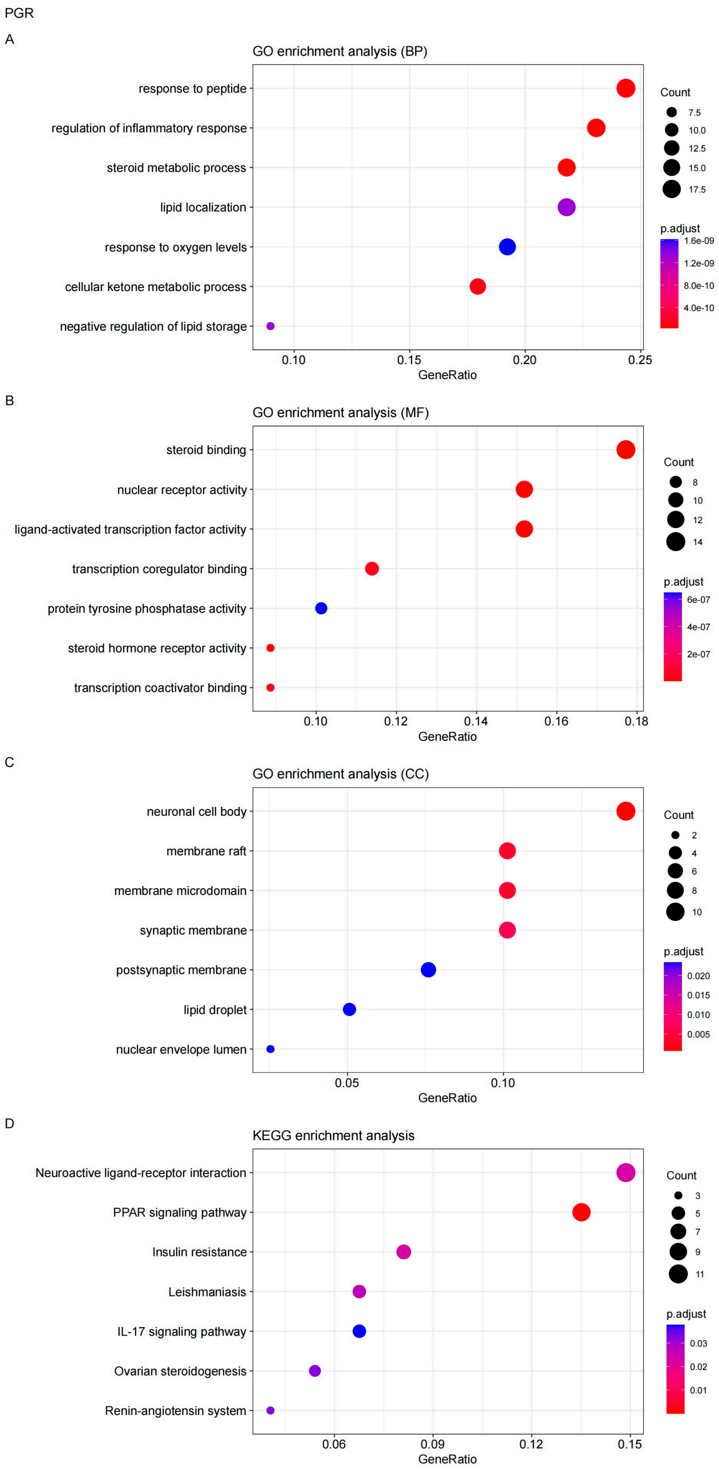

Figure S11.

The results of the GO and KEGG pathway enrichment analyses are visualized, with the size of the bubbles representing the number of genes. The GO analysis focuses on genes associated with biological processes, molecular functions, and cellular components related to Hederagenin and obesity. Meanwhile, the KEGG pathway enrichment analysis emphasizes the enriched pathways.

As shown in Figure S11(A), the GO and KEGG pathway enrichment analyses revealed several potential biological processes associated with Hederagenin, including response to peptide, regulation of inflammatory response, steroid metabolic process, lipid localization, response to oxygen levels, cellular ketone metabolic process, and negative

regulation of lipid storage.

Additionally, as shown in Figure S11 (B), the molecular functions identified through the GO analysis include steroid binding, nuclear receptor activity, ligand-activated transcription factor activity, transcription coregulator binding, protein tyrosine phosphatase activity, steroid hormone receptor activity, and transcription coactivator binding.

As shown in Figure S11 (C), the enriched cellular components identified include neuronal cell body, membrane raft, membrane microdomain, synaptic membrane, postsynaptic membrane, lipid droplet, and nuclear envelope lumen.

As shown in Figure S11 (D), the KEGG analysis identified enriched pathways that encompass the most critical targets, such as Neuroactive ligand-receptor interaction, PPAR signaling pathway, Insulin resistance, Leishmaniasis, IL-17 signaling pathway, Ovarian steroidogenesis, and Renin-angiotensin system.

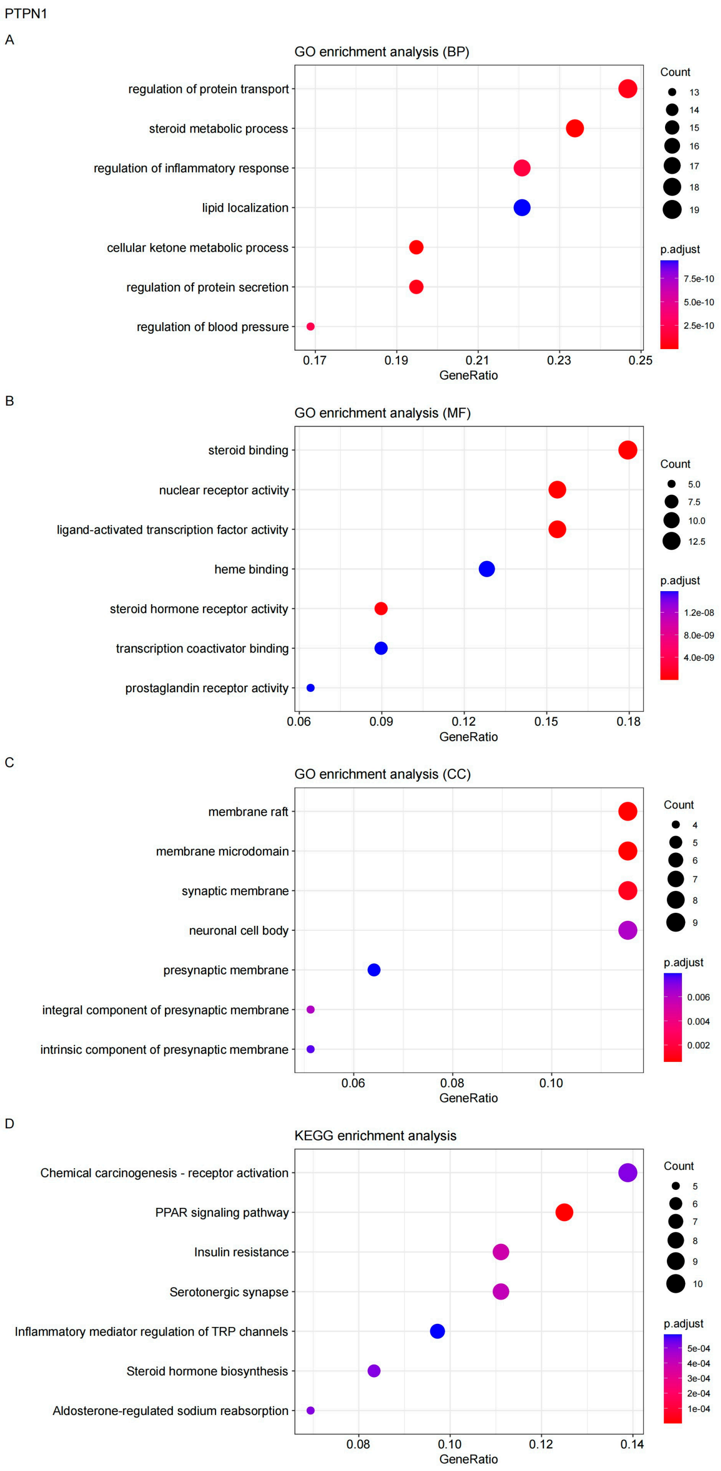

Figure S12.

The results of the GO and KEGG pathway enrichment analyses are visualized, with the size of the bubbles

representing the number of genes. The GO analysis focuses on genes associated with biological processes, molecular functions, and cellular components related to Triptonide and obesity. Meanwhile, the KEGG pathway enrichment analysis emphasizes the enriched pathways.

As shown in Figure S12(A), the GO and KEGG pathway enrichment analyses revealed several potential biological processes associated with Triptonide, including regulation of protein transport, steroid metabolic process, regulation of inflammatory response, lipid localization, cellular ketone metabolic process, regulation of protein secretion, and regulation of blood pressure.

Additionally, as shown in Figure S12(B), the molecular functions identified through the GO analysis include steroid binding, nuclear receptor activity, ligand-activated transcription factor activity, heme binding, steroid hormone receptor activity, transcription coactivator binding, and prostaglandin receptor activity.

As shown in Figure S12(C), the enriched cellular components identified include membrane raft, membrane microdomain, synaptic membrane, neuronal cell body, presynaptic membrane, integral component of presynaptic membrane, and intrinsic component of presynaptic membrane.

As shown in Figure S12(D), the KEGG analysis identified enriched pathways that encompass the most critical targets, such as Chemical carcinogenesis-receptor activation, PPAR signaling pathway, Insulin resistance, Serotonergic synapse, Inflammatory mediator regulation of TRP channels, Steroid hormone biosynthesis, and Aldosterone-regulated sodium reabsorption.

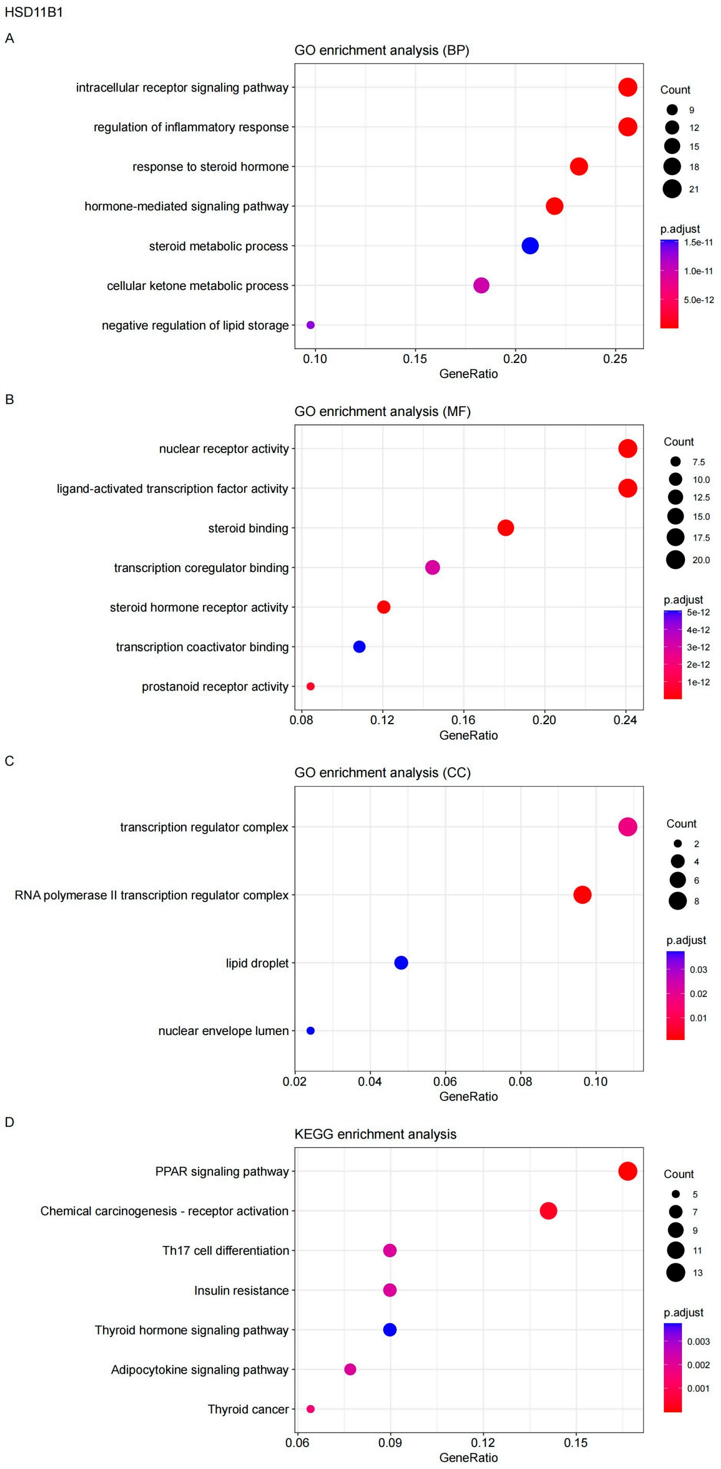

Figure S13.

The results of the GO and KEGG pathway enrichment analyses are visualized, with the size of the bubbles representing the number of genes. The GO analysis focuses on genes associated with biological processes, molecular functions, and cellular components related to Triptotriterpenic Acid B and obesity. Meanwhile, the KEGG pathway enrichment analysis emphasizes the enriched pathways.

As shown in Figure S13(A), the GO and KEGG pathway enrichment analyses revealed several potential biological processes associated with Triptotriterpenic Acid B, including the intracellular receptor signaling pathway, regulation of inflammatory response, response to steroid hormone, hormone-mediated signaling pathway, steroid metabolic process, cellular ketone metabolic process, and negative regulation of lipid storage.

Additionally, as shown in Figure S13(B), the molecular functions identified through the GO analysis include nuclear receptor activity, ligand-activated transcription factor activity, steroid binding, transcription coregulator binding, steroid hormone receptor activity, transcription coactivator binding, and prostanoid receptor activity.

As shown in Figure S13(C), the enriched cellular components identified include the transcription regulator

complex, RNA polymerase II transcription regulator complex, lipid droplet, and nuclear envelope lumen.

As shown in Figure S13(D), the KEGG analysis identified enriched pathways containing the most critical targets, such as the PPAR signaling pathway, Chemical carcinogenesis-receptor activation, Th17 cell differentiation, Insulin resistance, Thyroid hormone signaling pathway, Adipocytokine signaling pathway, and Thyroid cancer.

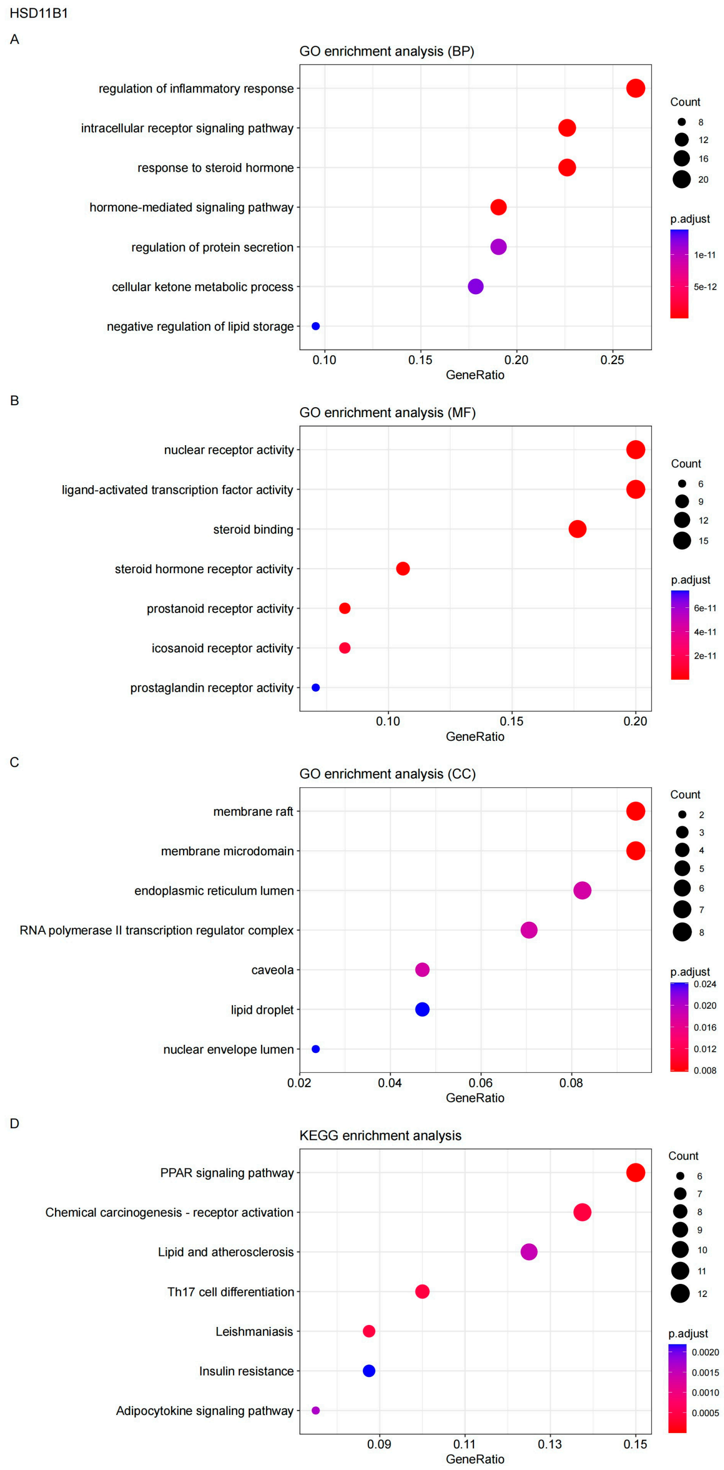

Figure S14.

The results of the GO and KEGG pathway enrichment analyses are visualized, with the size of the bubbles representing the number of genes. The GO analysis focuses on genes associated with biological processes, molecular functions, and cellular components related to Triptotriterpenic Acid C and obesity. Meanwhile, the KEGG pathway enrichment analysis emphasizes the enriched pathways.

As shown in Figure S14(A), the GO and KEGG pathway enrichment analyses revealed several potential biological processes associated with Triptotriterpenic Acid C, including regulation of inflammatory response, intracellular receptor signaling pathway, response to steroid hormone, hormone-mediated signaling pathway, regulation of

protein secretion, cellular ketone metabolic process, and negative regulation of lipid storage.

Additionally, as shown in Figure S14(B), the molecular functions identified through the GO analysis include nuclear receptor activity, ligand-activated transcription factor activity, steroid binding, steroid hormone receptor activity, prostanoid receptor activity, icosanoid receptor activity, and prostaglandin receptor activity.

As shown in Figure S14(C), the enriched cellular components identified include membrane raft, membrane microdomain, endoplasmic reticulum lumen, RNA polymerase II transcription regulator complex, caveola, lipid droplet, and nuclear envelope lumen.

As shown in Figure S14(D), the KEGG analysis identified enriched pathways containing the most critical targets, such as the PPAR signaling pathway, Chemical carcinogenesis-receptor activation, Lipid and atherosclerosis, Th17 cell differentiation, Leishmaniasis, Insulin resistance, and Adipocytokine signaling pathway.

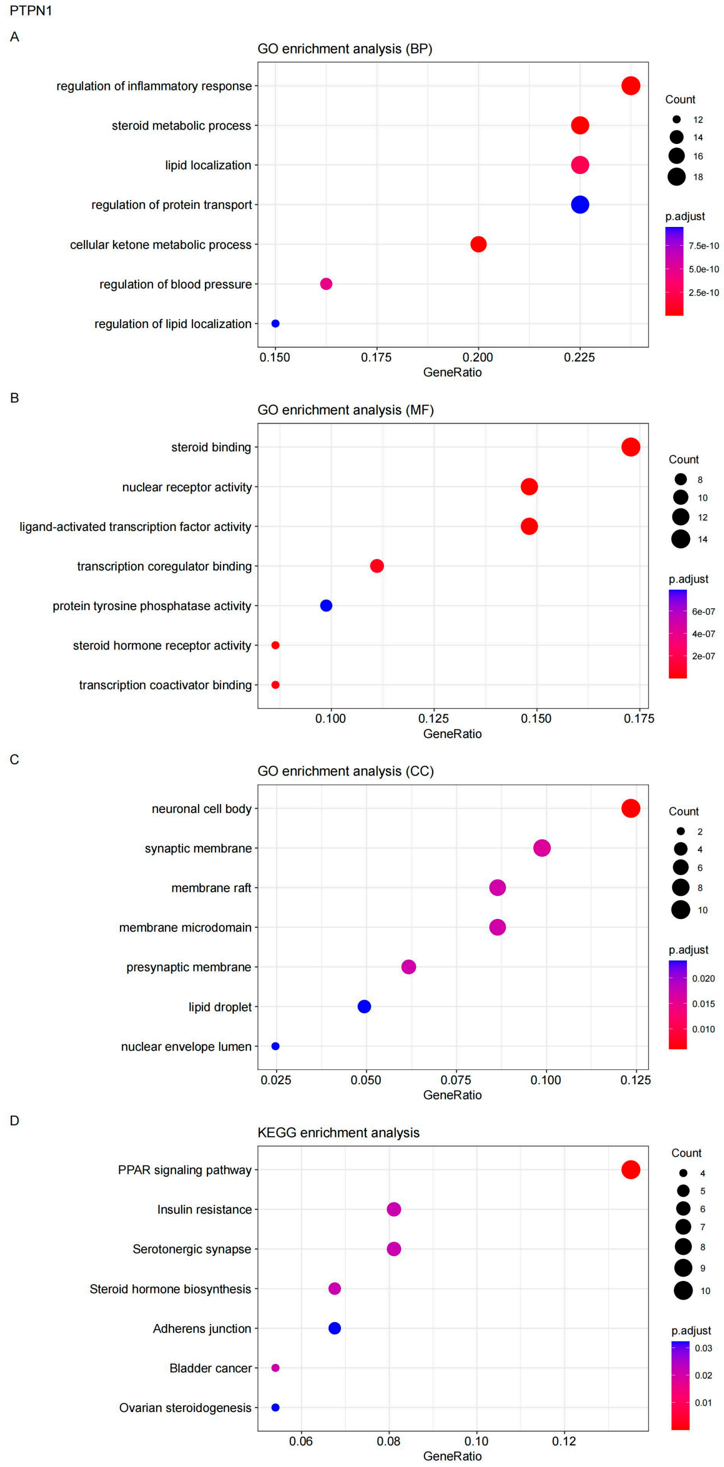

Figure S15.

The results of the GO and KEGG pathway enrichment analyses are visualized, with the size of the bubbles representing the number of genes. The GO analysis focuses on genes related to biological processes, molecular

functions, and cellular components associated with Ursolic Acid and obesity. Meanwhile, the KEGG pathway enrichment analysis highlights the enriched pathways.

As shown in Figure S15(A), the GO and KEGG pathway enrichment analyses revealed several potential biological processes associated with Ursolic Acid, including regulation of inflammatory response, steroid metabolic process, lipid localization, regulation of protein transport, cellular ketone metabolic process, regulation of blood pressure, and regulation of lipid localization.

Additionally, as shown in Figure S15(B), the molecular functions identified through the GO analysis include steroid binding, nuclear receptor activity, ligand-activated transcription factor activity, transcription coregulator binding, protein tyrosine phosphatase activity, steroid hormone receptor activity, and transcription coactivator binding.

As shown in Figure S15(C), the enriched cellular components identified include neuronal cell body, synaptic membrane, membrane raft, membrane microdomain, presynaptic membrane, lipid droplet, and nuclear envelope lumen.

As shown in Figure S15(D), the KEGG analysis identified enriched pathways containing the most critical targets, such as the PPAR signaling pathway, Insulin resistance, Serotonergic synapse, Steroid hormone biosynthesis, Adherens junction, Bladder cancer, and Ovarian steroidogenesis.

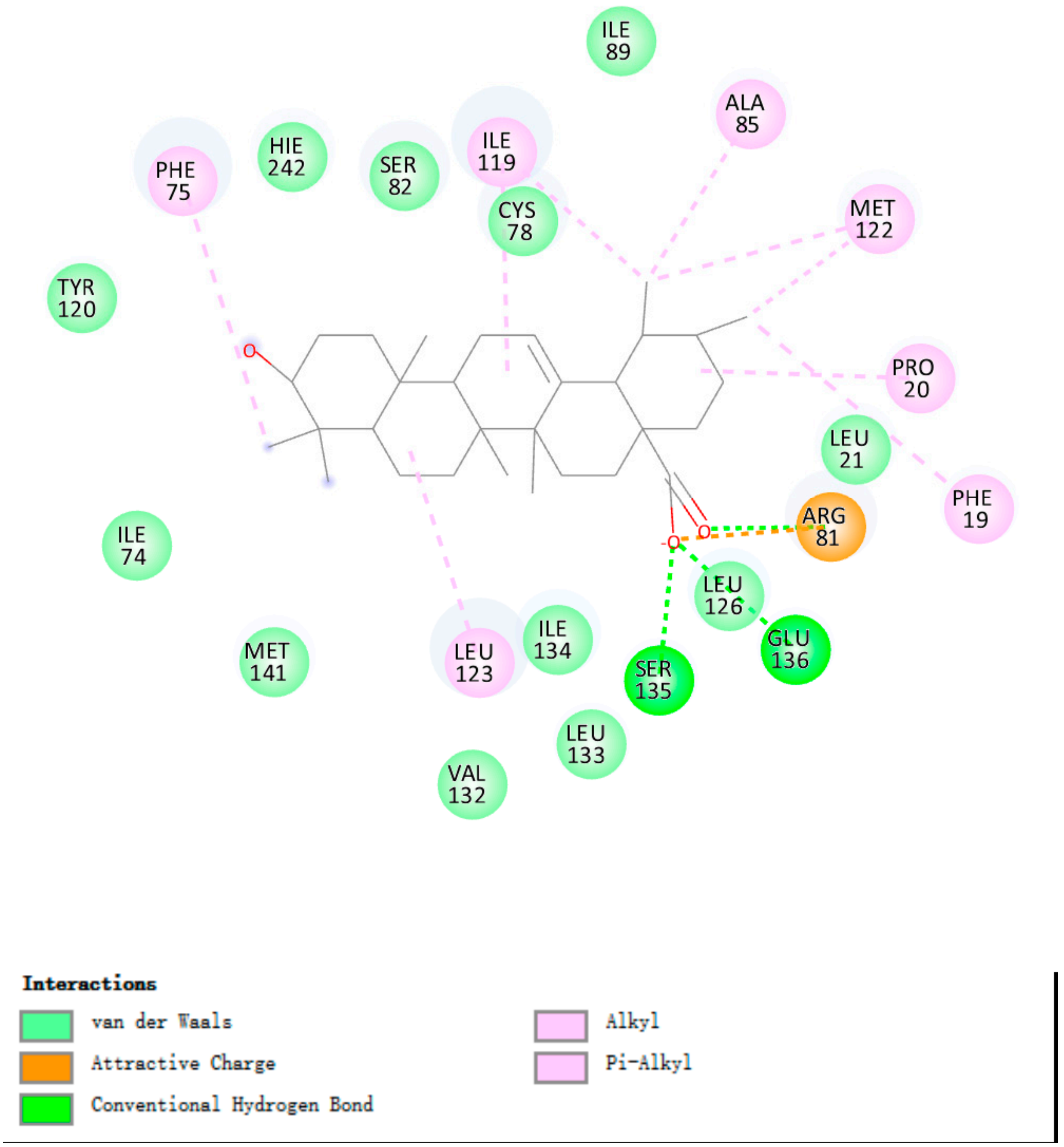

Figure S16 2D representation of the interaction forces between the Hederagenin and PPARG system

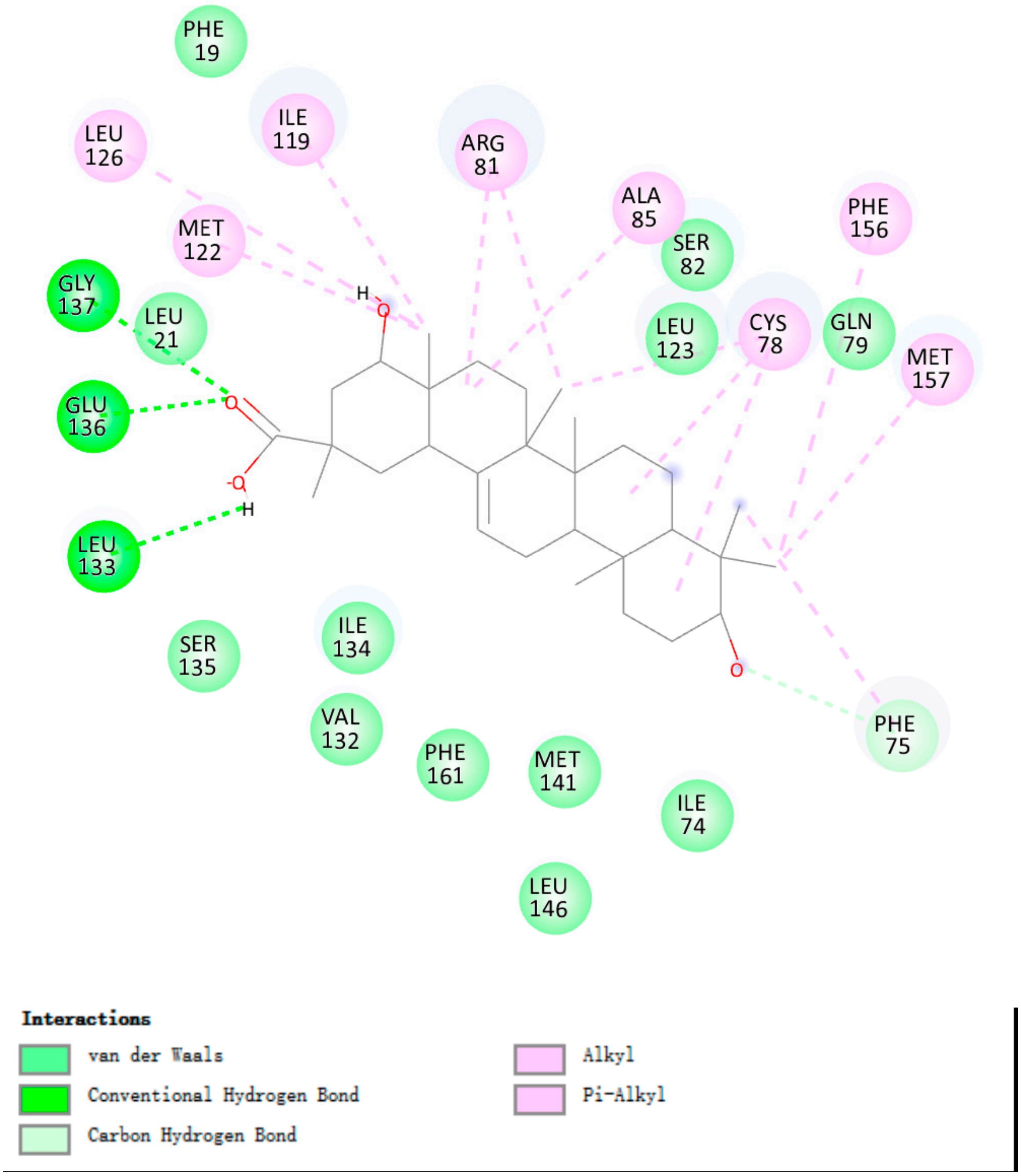

Figure S17 2D representation of the interaction forces between the Triptotriterpenic Acid B and PPARG system

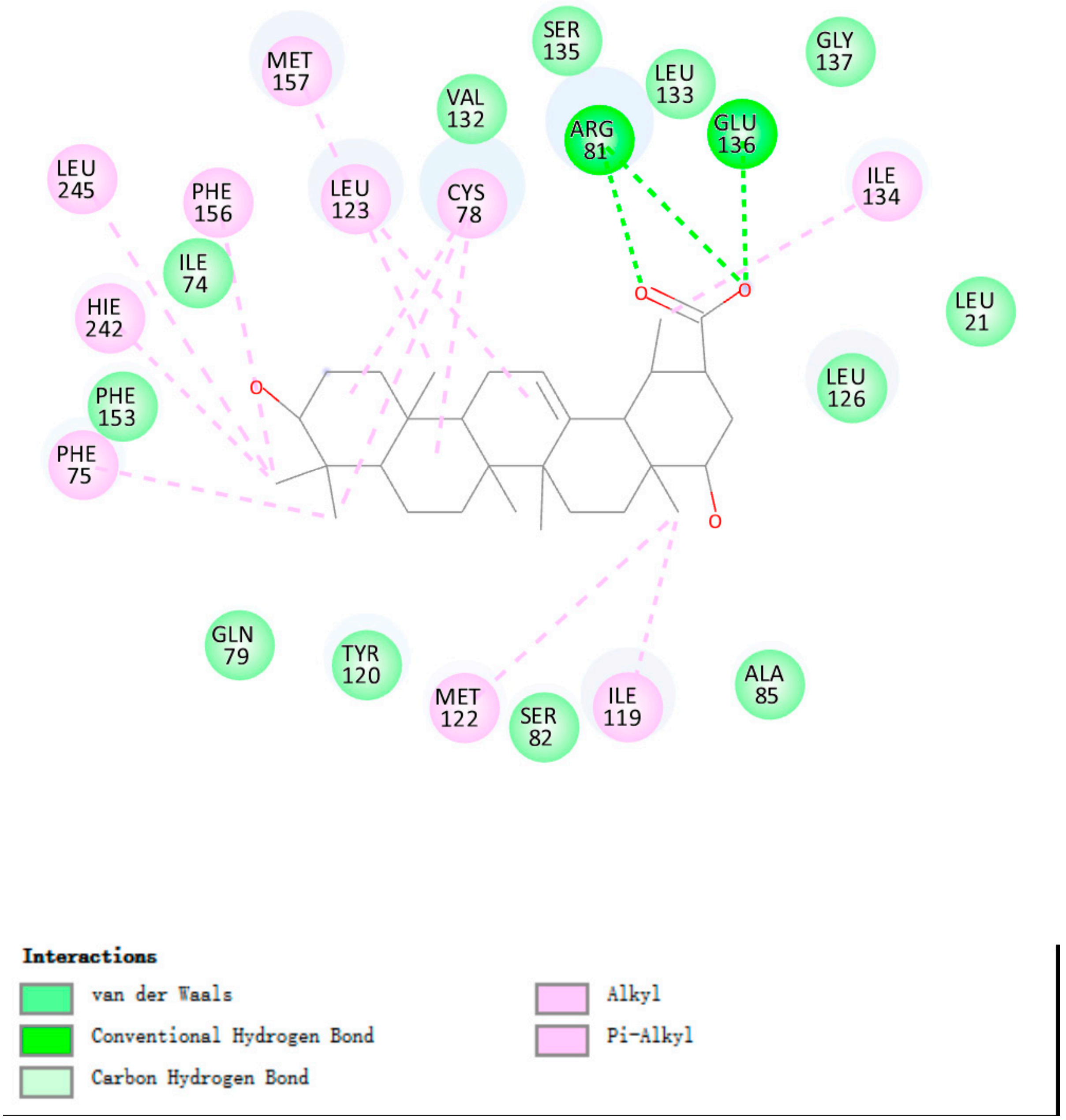

Figure S18 2D representation of the interaction forces between the Triptotriterpenic Acid C and PPARG system

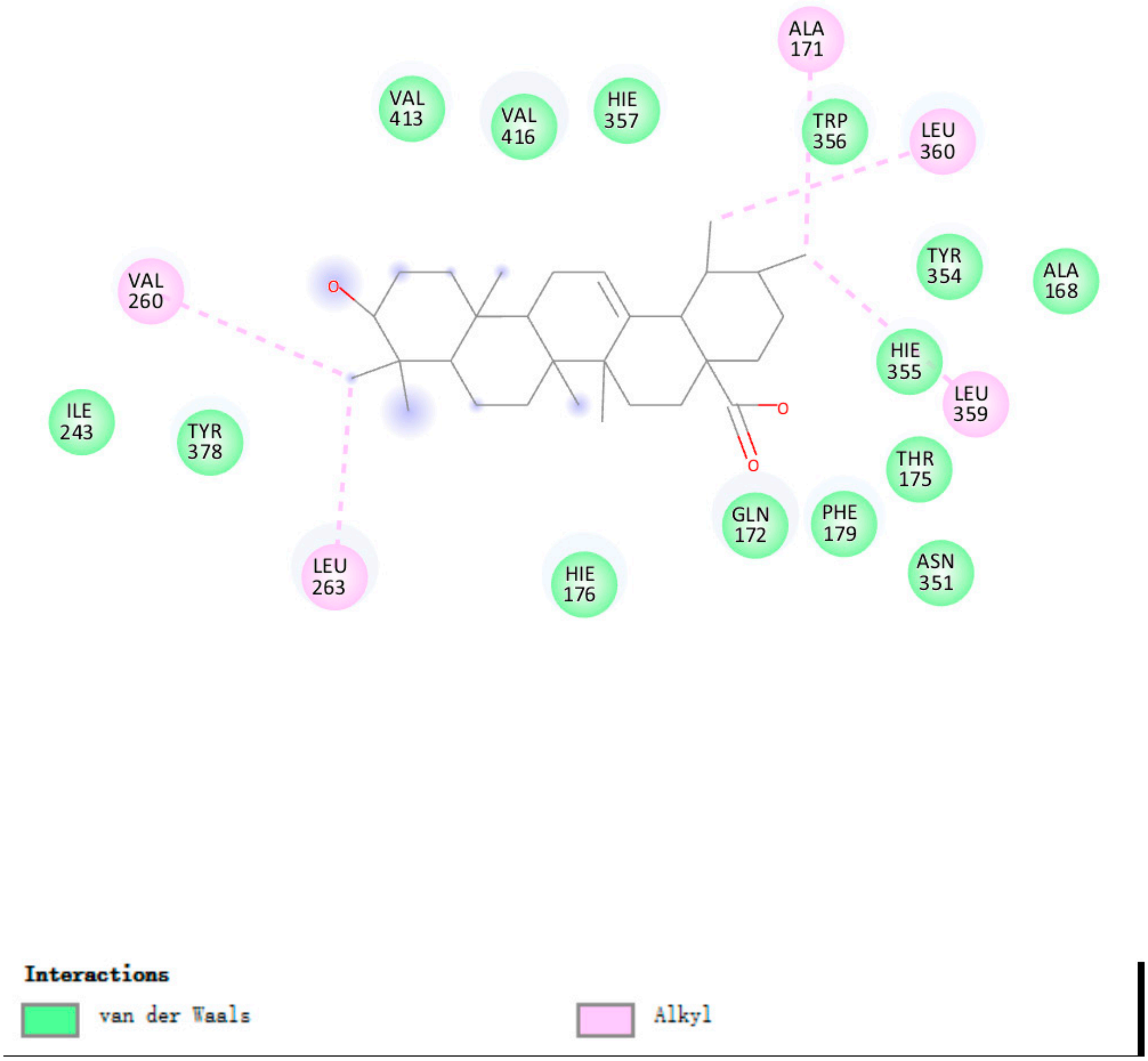

Figure S19 2D representation of the interaction forces between the Ursolic Acid and PTGS2 system

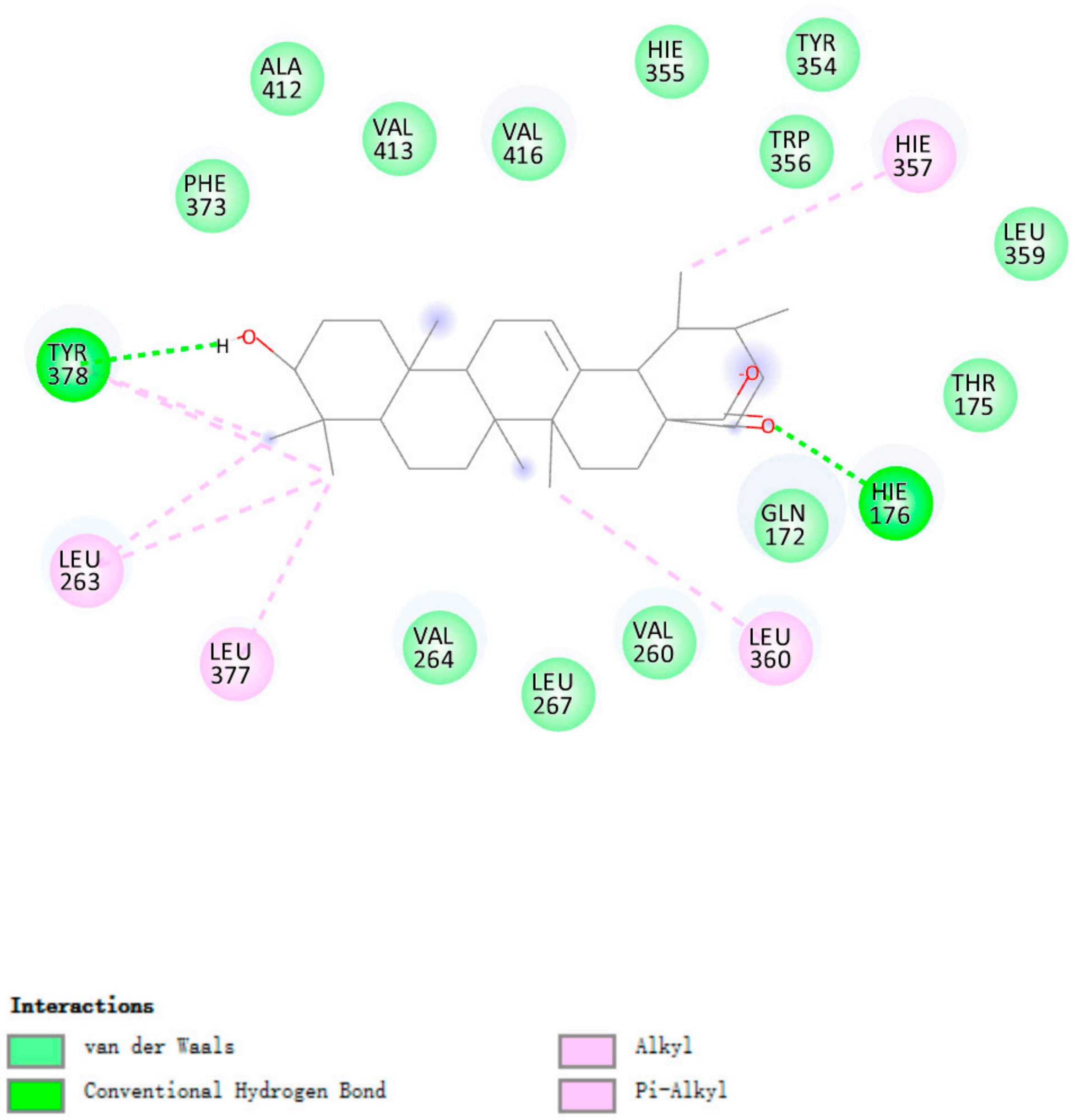

Figure S20 2D representation of the interaction forces between the Hederagenin and PTGS2 system

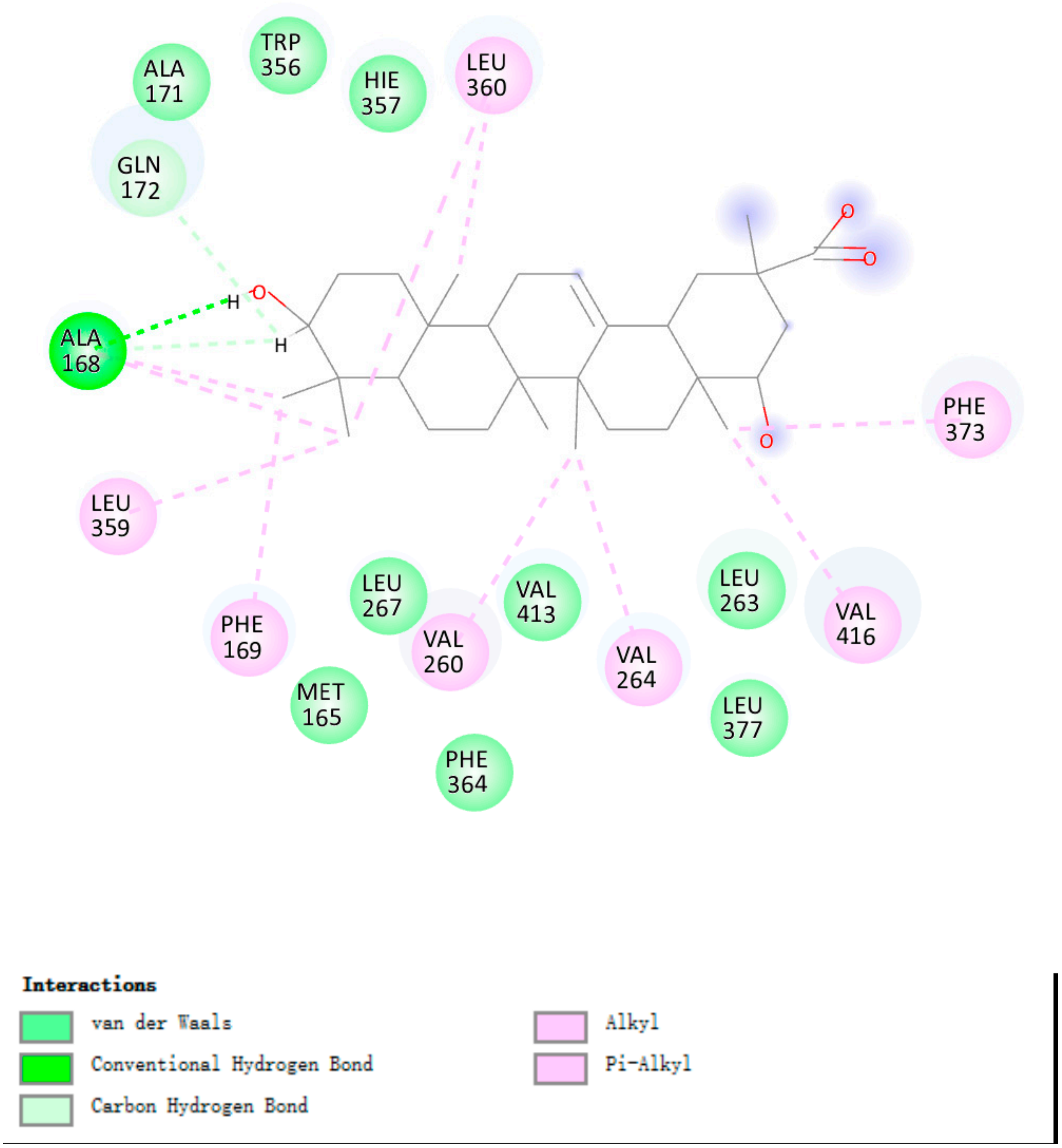

Figure S21 2D representation of the interaction forces between the Triptotriterpenic Acid B and PTGS2 system
