# Supplementary material for: Unveiling the Anti-Obesity Potential of Thunder God Vine: Network Pharmacology and Computational Insights into Celastrol-like Molecules
Source: Int J Mol Sci. 2024 Nov 21;25(23):12501. doi: 10.3390/ijms252312501 (PMC11641352; doi:10.3390/ijms252312501)
Supplement: Supplementary file 1 [file ijms-25-12501-s001.zip › ijms-3322425-supplementary/Supplementary/Supplementary Tables.pdf]

Table S1. Average hydrogen bond Results for Three Small Molecules with PPARG

System	Average hydrogen bond
Hederagenin	*2.06*
Triptotriterpenic Acid B	*0.411*
Triptotriterpenic Acid C	*1.419*

Table S2. Average hydrogen bond Results for Three Small Molecules with PTGS2

System	Average hydrogen bond
Hederagenin	*0.671*
Triptotriterpenic Acid B	*0.949*
Ursolic Acid	*0.384*

Table S3. Average hydrogen bond Results for Three Small Molecules with PPARG

System	No.	donor	acceptor	occupancy
Hederagenin	*1*	*ARG81-Side*	*6Q5271-Side*	*92.50%*
	2	*GLU136-Main*	*6Q5271-Side*	*71.30%*
	3	*SER135-Main*	*6Q5271-Side*	*15.70%*
Triptotriterpenic Acid B	*1*	*GLY137-Main*	*UNK271-Main*	*11.60%*
	2	*GLU136-Main*	*UNK271-Side*	*5.20%*
	3	*UNK271-Side*	*LEU133-Main*	*4.70%*
Triptotriterpenic Acid C	*1*	*UNK271-Side*	*GLU136-Side*	*67.20%*
	2	*GLU136-Main*	*UNK271-Main*	*35.40%*
	3	*GLU136-Main*	*UNK271-Side*	*16.10%*

Table S4. Average hydrogen bond Results for Three Small Molecules with PTGS2

System	No.	donor	acceptor	occupancy
Hederagenin	*1*	*HIE176-Side*	*6Q5552-Side*	*32.80%*
	2	*TYR378-Side*	*6Q5552-Side*	*23.60%*
	3	*6Q5552-Side*	*TYR378-Side*	*6.20%*
Triptotriterpenic Acid B	*1*	*UNK552-Side*	*ALA168-Main*	*59.30%*
	2	*UNK552-Side*	*ASN351-Side*	*9.00%*
	3	*UNK552-Side*	*GLN172-Side*	*7.30%*
Ursolic Acid	*1*	*UNK552-Side*	*TYR354-Main*	*17.90%*
	2	*UNK552-Side*	*THR175-Side*	*4.80%*
	3	*ASN351-Side*	*UNK552-Side*	*3.40%*
